# Development of Quality Control Ranges for Biocide Susceptibility Testing

**DOI:** 10.3390/pathogens11020223

**Published:** 2022-02-08

**Authors:** Angela R. Schug, Anissa D. Scholtzek, John Turnidge, Marita Meurer, Stefan Schwarz, Andrea T. Feßler, the Biocide Susceptibility Study Group

**Affiliations:** 1 Institute of Microbiology and Epizootics, Centre for Infection Medicine, Department of Veterinary Medicine, Freie Universität Berlin, 14163 Berlin, Germany; a.schug@posteo.de (A.R.S.); anissa.scholtzek@bfr.bund.de (A.D.S.); stefan.schwarz@fu-berlin.de (S.S.); 2 Veterinary Centre for Resistance Research (TZR), Freie Universität Berlin, 14163 Berlin, Germany; 3 Unit Bacterial Toxins, Food Service, Department Biological Safety, German Federal Institute for Risk Assessment, 10589 Berlin, Germany; 4 School of Biological Sciences, University of Adelaide, Adelaide, SA 5005, Australia; jturnidge@gmail.com; 5 Institute for Biochemistry, University of Veterinary Medicine Hannover, 30559 Hannover, Germany; marita.meurer@tiho-hannover.de; 6 Research Center for Emerging Infections and Zoonoses (RIZ), University of Veterinary Medicine Hannover, 30559 Hannover, Germany

**Keywords:** *Staphylococcus aureus*, *Enterococcus hirae*, *Escherichia coli*, *Pseudomonas aeruginosa*, benzalkonium chloride, chlorhexidine, octenidine, polyhexanide, minimal inhibitory concentration (MIC), interlaboratory trial

## Abstract

Every laboratory test needs validation by quality controls. For biocide susceptibility testing (BST), neither quality control (QC) strains nor QC ranges applicable to these strains are currently available. As QC strains, four well-defined laboratory reference strains (*Staphylococcus aureus* ATCC^®^ 6538, *Enterococcus hirae* ATCC^®^ 10541, *Escherichia coli* ATCC^®^ 10536 and *Pseudomonas aeruginosa* ATCC^®^ 15442), which have been used previously for biocide efficacy testing, were selected. In an interlaboratory trial with eleven participating laboratories, BST QC ranges should be developed for the aforementioned four strains and the four biocides benzalkonium chloride, chlorhexidine, octenidine and polyhexanide. The performance of three different lots of tryptic soy broth was explored using the broth microdilution method and the data were subsequently evaluated using the RangeFinder software. As a result, QC ranges were defined for all reference strain–biocide combinations, except for *P. aeruginosa* ATCC^®^ 15442 with the two biocides chlorhexidine and polyhexanide. The development of the latter two QC ranges was not possible, due to the limited solubility of the biocides in the test range required for *P. aeruginosa* ATCC^®^ 15442. The newly developed QC ranges comprise three to five dilution steps. The establishment of QC ranges will contribute to the validation of BST in the future.

## 1. Introduction

During recent years, not only an increase in antimicrobial resistance, but also an increase in resistance to biocides has been observed [1,2]. This is a challenge to veterinary and public health that corroborates the challenges posed by antimicrobial resistance [2]. Different classes of biocides are widely used, such as quaternary ammonium compounds (e.g., benzalkonium chloride), biguanides (e.g., chlorhexidine and polyhexanide [polyhexamethylene biguanide hydrochloride]), bispyridines (e.g., octenidine [octenidine dihydrochloride]), aldehydes (e.g., glutardialdehyde) and alcohols (e.g., ethanol, isopropanol). Biocide susceptibility testing (BST) is an important tool for the determination of phenotypic biocide susceptibility, which allows surveillance and monitoring of the emergence and prevalence of biocide resistance of bacterial pathogens that are of significance for public health as well as veterinary and human medicine. Standardized protocols have long been established for biocide efficacy testing (BET) [3,4], but no harmonized protocols for BST were available for a long time. During recent years, BST protocols for broth macrodilution [5] and broth microdilution [6] have been developed. During the evaluation of different parameters, including inoculum preparation and different incubation times, these methods seemed to be rather robust [5,6].

Nevertheless, it is important to have an internal control system for any technical method. Approved ranges for quality control (QC) strains are essential when testing bacteria for their susceptibility to antimicrobial agents to ensure test performance and reliable test results [7,8]. Such QC ranges were developed for defined QC strains of different bacterial species, e.g., *Staphylococcus aureus* ATCC^®^ 29213, *S. aureus* ATCC^®^ 25923 or *Escherichia coli* ATCC^®^ 25922, and published in the documents issued by, e.g., the CLSI and the EUCAST [9,10,11]. The testing of these QC strains together with the test strains is required to allow a validation of the test system and obtain reliable results, since there are many factors including technical expertise, media composition, pH value, inoculum densities, and incubation parameters, which might influence the test results [7,8,10]. The same is true for the validation of BST, where QC ranges are also required to validate the test performance. However, so far, there are no QC ranges for reference strains used in broth dilution BST methods [5,6]. Therefore, our aim was to establish QC ranges for the four reference strains used for the development of the broth microdilution method, namely *S. aureus* ATCC^®^ 6538, *Enterococcus hirae* ATCC^®^ 10541, *E. coli* ATCC^®^ 10536 and *Pseudomonas aeruginosa* ATCC^®^ 15442 [6]. Moreover, these reference strains are generally used for BET [3,4]. The selection of biocides for the QC range development should represent different classes of biocides and the aim was to use commercially available microtiter plates. For this purpose, the biocides must be stable in their activity over a longer period of time in a dry state in the 96 well plate. Therefore, the alcohols, being represented by isopropanol in previous studies [5,6] were excluded. According to the manufacturer of the microtiter plates, technical reasons prevented the inclusion of glutardialdehyde in the custom-made microtiter plates. Finally, four biocides from three different classes, namely benzalkonium chloride, chlorhexidine, octenidine and polyhexanide, were chosen for QC range development during an interlaboratory trial. These biocides are widely used in different areas such as infection prevention and hygiene, in health care facilities, households and in the food industry [1,12,13].

## 2. Results and Discussion

### 2.1. Interlaboratory Trial

In total, eleven laboratories (lab 1 to lab 11) from Germany participated in this study and tested the four reference strains which were chosen as future QC strains. Each of the reference strains—*S. aureus* ATCC^®^ 6538, *E. hirae* ATCC^®^ 10541, *E. coli* ATCC^®^ 10536, and *P. aeruginosa* ATCC^®^ 15442—was tested ten times with three different media lots (lots A to C) for their susceptibility to the four biocides benzalkonium chloride, chlorhexidine, octenidine and polyhexanide.

Each laboratory provided 30 data points for each biocide–reference strain combination, including ten data points for each of the three media lots. This resulted in 330 data points for each of the 16 biocide–reference strain combinations that were included in the calculation of the QC ranges. The bacterial cell counts, determined in parallel for each test, were all within the acceptable range of 1 × 10^8^–1 × 10^9^ CFU/mL, which are required to assure standardized inoculum densities and test conditions [5,6,14].

For the development of new QC ranges for AST, a related substance with an already established QC range should be included, which is tested in parallel to the substance [15]. However, this was not possible in the present study, since there were no QC ranges at all available for BST.

### 2.2. Development of QC Ranges for Staphylococcus aureus ATCC^®^ 6538

The reference strain *S. aureus* ATCC^®^ 6538, also known as *S. aureus* DSM 799, was chosen, since it has been used for a long time for BET [3,4] and was also used for the establishment of BST via broth macrodilution [5] and broth microdilution [6].

#### 2.2.1. Benzalkonium Chloride

For *S. aureus* ATCC^®^ 6538 and benzalkonium chloride, the minimal inhibitory concentrations (MICs) ranged from 0.00003% to 0.001% including six dilution steps. Results for lots A and C ranged over three dilution steps each, and those from lot B over five dilution steps (Figure S1a). Regarding lot B, it should be mentioned that all but three MIC values with 0.001% were within two dilution steps (0.00006% and 0.000125%). In total, 322 MIC values (97.58%) were within these two dilution steps (Figure S1a).

The RangeFinder software was used to calculate a QC range for BST with *S. aureus* ATCC^®^ 6538 and benzalkonium chloride, and revealed a QC range of 0.00003–0.00025%, including four dilution steps. In total, 327 of the 330 MIC values are included in this QC range, which represents 99.1% of the values (Table 1). The development of a QC range of four dilution steps is in accordance with the CLSI standards for AST [15].

The results are in accordance with the data from previous studies, which revealed similar MICs of 0.00005% to 0.0004%, during the establishment of the broth macrodilution protocol [5] and ≤0.000025% to 0.0004% during the interlaboratory trial associated with the development of this method [5]. During the establishment of the broth microdilution method, benzalkonium chloride MICs of 0.00006% and 0.00025% were observed, which is in accordance with the present study [6]. The similarity of the results from the method development and the interlaboratory trial also showed that it is possible to use commercial microtiter plates in which benzalkonium chloride is present in dried form.

#### 2.2.2. Chlorhexidine

The MIC values for *S. aureus* ATCC^®^ 6538 and chlorhexidine comprised four dilution steps (0.00003–0.00025%). The media lots A, B and C comprised three, four and four dilution steps, respectively (Figure S1b). During establishment of the broth macrodilution protocol, similar MICs in the range between 0.00005% and 0.0002% were observed [5].

The MICs of 0.00006% and 0.000125% observed during the establishment of the broth microdilution method are within the MIC range observed during this study [6]. The similarity of the results from the method development and the interlaboratory trial confirms the possibility to use commercial microtiter plates with chlorhexidine in dried form.

The interlaboratory comparison revealed that all but one laboratory (lab 4) had MIC values in the range of 0.00003–0.000125%. In addition to lab 4, only labs 2 and 5 showed MIC distributions over three dilution steps. The values of the remaining laboratories included only two dilution steps (Table 2). These results point towards good reproducibility and comparability between different laboratories. Based on the results, a QC range of 0.00003–0.00025% was determined, which included all values (100.0%) (Table 2).

#### 2.2.3. Polyhexanide

For *S. aureus* ATCC^®^ 6538 and polyhexanide, a comparatively wide MIC distribution was observed including the same six dilution steps from 0.00006% to 0.002% for all three media lots. The majority of the MIC values was seen within the same three dilution steps (0.000125–0.0005%) (Figure S1c). Overall, the three media lots showed comparable results.

The RangeFinder software calculated a QC range from 0.00006% to 0.001% including five dilution steps (Table 3). This is a rather large QC range, compared to CLSI recommendations for AST, suggesting three or in maximum four dilution steps [15]. Even though the proposed QC range includes five dilution steps, five values (originating from two different laboratories) were outside the acceptable range. Therefore, 325 values (98.5%) are within this QC range.

The interlaboratory comparison showed differences regarding the MIC distributions. Two laboratories had MIC distributions over two (labs 3 and 7), three laboratories over three (labs 1, 5, and 10), five laboratories over four (labs 2, 4, 8, 9, and 11) and the remaining lab 6 over six dilution steps. In the latter one, two values were at least +3 dilution steps higher than the remaining MICs obtained in this laboratory. Moreover, between the laboratories, some differences were observed, which resulted in a distribution of six dilution steps. Some laboratories determined mainly lower MICs, some determined MICs more in the middle of the range and other laboratories identified wider distributions including also higher MICs (Table 3).

#### 2.2.4. Octenidine

BST of *S. aureus* ATCC^®^ 6538 with octenidine revealed a MIC distribution over five dilution steps (0.00003–0.0005%). Four, four and three dilution steps were observed for the media lots A, B and C, respectively. Medium lot A revealed slightly lower MIC values compared with the MICs obtained by using the lots B and C (Figure S1d). Only single values displayed MICs of 0.00003% (lot A) and 0.0005% (lot B).

Having a look at the interlaboratory agreement, only two laboratories had MIC distributions over four dilution steps, lab 1 with one “higher” value of 0.0005% and lab 6 with one “lower” MIC of 0.00003%. Lab 11 showed an equal distribution of the values over two dilution steps, and the remaining laboratories included the three dilution steps 0.00006%, 0.000125% and 0.00025% (Table 4).

Based on the results obtained in the eleven laboratories, it is not surprising that the RangeFinder software identified the three dilutions from 0.00006% to 0.00025% with 328 included values (99.4%) as the QC range (Table 4).

#### 2.2.5. Summary *Staphylococcus aureus* ATCC^®^ 6538

For *S. aureus* ATCC^®^ 6538 QC, ranges could be determined for all four biocides tested (Table S1). For octenidine, the MIC distributions seemed to be very stable and resulted in QC ranges covering three dilution steps (see Section 2.2.4). In comparison, for benzalkonium chloride, a four dilution step QC range was determined, even though only one single value was found in the lowest dilution step of this QC range (0.00003%) (see Section 2.2.1). The reason for including this dilution step is that 195 of the 330 values had a MIC of 0.0006% and hence, this is the mode value. Therefore, the dilution step of 0.00003% was included. In addition, for chlorhexidine, a four dilution step QC range was determined including all identified MIC values (see Section 2.2.2). In the case of polyhexanide, the results were more diverse and consequently resulted in a broader QC range including five dilution steps (see Section 2.2.3).

### 2.3. Development of QC Ranges for Enterococcus hirae ATCC^®^ 10541

For BET, the reference strain *E. hirae* ATCC^®^ 10541, also known as *E. hirae* DSM 3320, is commonly used [3,4] and hence was also chosen for the establishment of the broth microdilution BST method [6]. Moreover, it was also included in an interlaboratory trial using the broth macrodilution method for BST [5]. Therefore, the strain is well established for the different types of biocide susceptibility testing and was selected as QC strain for the development of QC ranges in the present study.

#### 2.3.1. Benzalkonium Chloride

The MIC distribution for *E. hirae* ATCC^®^ 10541 and benzalkonium chloride included three dilution steps, ranging from 0.000125% to 0.0005%. While medium lot A comprised only two dilution steps, the remaining two lots comprised tree dilution steps, however with only single values with a MIC of 0.0005% (Figure S2a). This means that 328 values (99.4%) are included within only two dilution steps, indicating a good agreement between the media lots. During an interlaboratory trial for broth macrodilution, MIC values of ≤0.00006% to 0.0005% were observed [5], which are similar to the results of this study. The results of this study are also in accordance with the previous study on the establishment of a broth microdilution method, which revealed MICs of 0.000125% to 0.0005% [6].

Regarding the interlaboratory comparison, seven laboratories only determined the two most common dilution steps. Lab 6 and lab 10 included all three dilution steps. In lab 7, all MICs were 0.000125%; and in lab 9, all MICs were 0.00025% (Table S2a).

The RangeFinder software calculated a QC range of 0.000125–0.0005% for *E. hirae* ATCC^®^ 10541 and benzalkonium chloride. This QC range comprised all 330 values (100%) obtained from the eleven laboratories with the three media lots tested (Table S2a). The performance of *E. hirae* ATCC^®^ 10541 and benzalkonium chloride was very stable and reproducible, both in the commercial microtiter plates and in previous tests with freshly prepared biocide solutions [5,6].

#### 2.3.2. Chlorhexidine

For *E. hirae* ATCC^®^ 10541 and chlorhexidine, the MICs were distributed over four dilution steps (0.00003–0.00025%). MIC determination revealed three, three and four dilution steps when using the media lots A, B and C, respectively. Only a single value of 0.00003% was detected for lot C (Figure S2b). The interlaboratory comparison showed that five laboratories each determined MICs comprising two or three dilution steps, while only lab 8 determined all four dilution steps (Table S2b).

A QC range of 0.00003–0.00025% was determined for *E. hirae* ATCC^®^ 10541 and chlorhexidine, including all 330 MIC values obtained during the interlaboratory trial (Table S2b). These results with all values within the four dilution step QC range point towards a good interlaboratory comparability and stability of this BST broth microdilution method. The similarity of the results from the method development [6] and the interlaboratory trial [5] also shows that it is possible to use commercial microtiter plates in which chlorhexidine is present in dried form.

#### 2.3.3. Polyhexanide

The testing of polyhexanide with *E. hirae* ATCC^®^ 10541 revealed a comparatively wide MIC distribution over seven dilution steps (0.000125–0.008%). A comparison of the media lots revealed, that six, five and four dilution steps were measured for lot A, lot B and lot C, respectively. Overall, the MIC distribution looks similar; however, lot A seems to provide slightly higher and lot B slightly lower MICs (Figure S2c). Interlaboratory comparisons showed some deviations, as two laboratories (lab 7, six dilution steps and lab 11, four dilution steps) determined MICs of ≥0.004%. The results of lab 8 comprised five dilution steps, and those of two laboratories, lab 1 and lab 10, three dilution steps. The remaining six laboratories displayed MIC distributions covering four dilution steps (Table S1c).

The QC range for *E. hirae* ATCC^®^ 10541 and polyhexanide includes five dilution steps and ranges from 0.000125% to 0.002% (Table S2c). Compared to the CLSI AST recommendations, this QC range is too wide. However, for the moment, we suggest this QC range, but consider a possible later reevaluation, when able to validate the test by comparative testing of another biocide with approved QC ranges.

#### 2.3.4. Octenidine

The MIC distribution for *E. hirae* ATCC^®^ 10541 and octenidine comprised five dilution steps (0.00006–0.001%). MIC determination using media lots A and B included four dilution steps (lot A 0.00006–0.0005% and lot B 0.000125–0.001%). Lot C included only three dilution steps (0.000125–0.0005%). Ten measurements revealed a MIC of 0.00006% (lot A) and two a MIC of 0.001% (lot B).

The interlaboratory comparison revealed that five laboratories (labs 1, 2, 6, 8, and 9) determined only MICs of the two dilution steps (0.000125% and 0.00025%). Lab 7 also measured two dilution steps (0.00006% and 0.000125%). Four laboratories (labs 3, 4, 5, and 10) determined MICs within a three dilution step range and lab 11 measured four dilution steps, including the two highest MIC values of 0.001% (Table S2d).

The RangeFinder software calculated a QC range of 0.00006–0.0005% for *E. hirae* ATCC^®^ 10541 and octenidine. This range includes four dilution steps with 328 MIC values (99.4%).

#### 2.3.5. Summary *Enterococcus hirae* ATCC^®^ 10541

In summary, only for polyhexanide and *E. hirae* ATCC^®^ 10541 a large QC range including five dilution steps was determined (see Section 2.3.3), which is more than the maximum of four dilution steps recommended by the CLSI for AST [15]. In comparison, the QC ranges for benzalkonium chloride (three dilution steps, see Section 2.3.1) as well as chlorhexidine (see Section 2.3.2) and octenidine (see Section 2.3.4) with four dilution steps each are more favorable (Table S3). These results allow a validation of BST with *E. hirae* ATCC^®^ 10541 as QC strain.

### 2.4. Development of QC Ranges for Escherichia coli ATCC^®^ 10536

*E. coli* ATCC^®^ 10536, also known as *E. coli* DSM 682, is also one of the reference strains used for BET [3,4] and hence was selected for BST establishment [6]. In addition, this strain was also included in a previous interlaboratory trial to investigate the performance of a broth macrodilution BST method [5]. Therefore, this reference strain was considered as stable and appropriate as QC strain for BST.

#### 2.4.1. Benzalkonium Chloride

For *E. coli* ATCC^®^ 10536 and benzalkonium chloride, the MICs ranged from 0.00025% to 0.002% including four dilution steps. The use of the media lots A, B and C resulted in MIC values comprising three, four and two dilution steps, respectively (Figure S3a). The three media lots yielded similar results. In total, 87 values displayed a MIC of 0.0005%, 217 values a MIC of 0.001% and 25 values a MIC of 0.002%. Only a single value with a MIC of 0.00025% was outside the range of the most common value +/− one dilution step (Table 5).

During a previous interlaboratory trial using broth macrodilution, MIC values of 0.001% to 0.004% were obtained [5], which were slightly higher compared to the present study. In addition, the results obtained during the establishment of the broth microdilution BST method are in accordance with the results of this study and revealed MICs of 0.0005% to 0.002% [6]. The similarity of the results from the method development and the interlaboratory trial also shows that it is possible to use commercial microtiter plates in which benzalkonium chloride is present in dried form.

Comparison of the laboratories revealed that only lab 6 had measured a single MIC value of 0.00025% included in a range of three dilution steps. Five other laboratories (labs 1, 3, 4, 9, and 11) had also determined MICs in a three dilution range, while the MIC distributions of the remaining laboratories comprised only two dilution steps (Table 5).

The establishment of the QC ranges with the RangeFinder software revealed a QC range of 0.0005–0.002%, comprising three dilution steps. This QC range includes 329 of the 330 included MICs, which represents 99.7% of the values (Table 5).

#### 2.4.2. Chlorhexidine

The chlorhexidine MICs for *E. coli* ATCC^®^ 10536 included a total of six dilution steps from 0.00003% to 0.001%. The use of the different media lots A, B and C resulted in six, five and four dilution steps (Figure S3b). All but five MIC values were within the four dilution ranges 0.00003% to 0.00025%. There were slight differences regarding the MIC distributions of the different media lots (Figure S3a), but they did not seem to have a larger influence on the determination of the QC ranges.

The MIC range of 0.00003–0.000125% obtained during the establishment of the broth microdilution method is covered by the MIC distribution observed during this study [6]. The similarity of the results from the method development and the interlaboratory trial [5] also shows that it is possible to use commercial microtiter plates in which chlorhexidine is present in dried form. The MIC distributions of the different laboratories comprised two (lab 7) to six dilution steps (lab 11) (Table 6).

A QC range of 0.000015–0.00025% (five dilution steps) was determined for *E. coli* ATCC^®^ 10536 and chlorhexidine using the RangeFinder software (Table 6). Six values were outside the acceptable range, which results in 98.2% of the values being included in the QC range. Interestingly, these five included dilution steps also contain an MIC value of 0.000015%. However, this MIC value was not determined during this interlaboratory trial, but has been included in the calculated range, probably due to the fact that 84 MIC values of 0.00003% were detected. The mean value was 0.00006% and was detected in 141 cases.

#### 2.4.3. Polyhexanide

BST for *E. coli* ATCC^®^ 10536 and polyhexanide revealed a MIC distribution from 0.00006% to ≥ 0.064% comprising at least eleven dilution steps. However, without the single MIC of ≥ 0.064%, the MIC distribution is reduced to the much more favorable six dilution steps (Figure S3c). Most of the values are within the three dilution steps from 0.000125% to 0.0005%, which include 311 values (94.2%). The use of all media lots revealed comparable MIC distributions. Therefore, an influence of the media lots tested was not observed. The different media lots comprised the aforementioned at least eleven (lot A), six (lot B) and five dilution steps (lot C) (Figure S3c).

Interlaboratory comparison showed mainly distributions over three to four dilution steps, sometimes a bit higher or lower, compared to other laboratories. Lab 6 had the most reproducible results, with 29 values of 0.000125% and only a single value of 0.00025%. In contrast, lab 11 displayed the widest range, with at least ten dilution steps included. This includes a single value of ≥ 0.064%, which is probably not valid. Without this value, the MIC distribution only includes five dilution steps, which is much more favorable (Table 7).

This wide MIC distribution resulted in a QC range of five dilution steps (0.00006–0.001%) for *E. coli* ATCC^®^ 10536 and polyhexanide. Five values, all from lab 11, were not included in the QC range. Overall, 325 of the 330 MIC values (98.5%) were included in this QC range.

#### 2.4.4. Octenidine

The testing of *E. coli* ATCC^®^ 10536 and octenidine revealed a MIC distribution over six dilution steps. The use of media lots A and C covered four dilution steps while that of lot B included all six dilution steps. The MIC distributions of the media lots showed slight differences (Figure S3d).

At the laboratory level, the MIC distributions ranged from two (lab 7) to six (lab 11) dilution steps. The results of five laboratories (labs 1, 2, 5, 6, and 9) included three dilution steps and those of the remaining four laboratories four dilution steps. Three laboratories (labs 3, 4 and 11) determined MIC values of ≥ 0.001% (Table 8).

The RangeFinder software calculated a QC range of 0.00006–0.0005%, which, therefore, includes four dilution steps. Seven MIC values were not included, resulting in 97.9% of the measured values being within this octenidine QC range for *E. coli* ATCC^®^ 10536 (Table 8).

#### 2.4.5. Summary *Escherichia coli* ATCC^®^ 10536

QC ranges for all four biocides were determined for *E. coli* ATCC^®^ 10536 (Table S4). Comparatively wide QC ranges of five dilution steps were determined for chlorhexidine and polyhexanide (Section 2.5.2 and Section 2.5.3), which both belong to the class of biguanides. For benzalkonium chloride (three dilution steps; see Section 2.5.1) and octenidine (four dilution steps; see Section 2.5.4), the QC ranges are in accordance with CLSI recommendations for AST [15].

### 2.5. Development of QC Ranges for Pseudomonas aeruginosa ATCC^®^ 15442

*P. aeruginosa* ATCC^®^ 15442, also known as *P. aeruginosa* DSM 939, is a reference strain, which is also commonly used for BET [3,4]. The strain was—as the previously described reference strains—selected as QC strain for BST, since it was used for BST broth microdilution establishment [6] and the broth macrodilution interlaboratory trial [5].

#### 2.5.1. Benzalkonium Chloride

The benzalkonium chloride MIC distribution for *P. aeruginosa* ATCC^®^ 15442 comprised at least five dilution steps, from 0.002% to ≥ 0.032%. The use of the different media lots A, B and C yielded MICs in ranges comprising at least four, three and two dilution steps. While media lots B and C showed very similar results, lot A seemed to produce slightly higher (one dilution step) MIC values (Figure S4a). However, this difference should be acceptable. All but two values were 0.004% or 0.008%. Lab 7 determined one value of 0.002% and lab 4 one value of ≥ 0.032% (Table S5a). Similar results with MICs from 0.002% to 0.016% were obtained in a broth macrodilution interlaboratory trial [5]. The similarity of the results from the method development and the interlaboratory trial also confirms that it is possible to use commercial microtiter plates in which benzalkonium chloride is present in dried form.

For *P. aeruginosa* ATCC^®^ 15442 and benzalkonium chloride, a QC range of 0.002–0.008% was determined using the RangeFinder software, comprising three dilution steps (Table S5a). In total, 329 MIC values (99.7%) were included, which means that only one value was outside the QC range.

#### 2.5.2. Chlorhexidine

For *P. aeruginosa* ATCC^®^ 15442, the chlorhexidine MIC distribution comprised at least six dilution steps and ranged from 0.0005% to ≥0.016%. At least five, five and six dilution steps were determined when using the media lots A, B and C, respectively. The different media lots showed comparable results (Figure S4b). At the laboratory level, the MIC distributions ranged from two (lab 8) to six dilution steps (lab 4) (Table S5b). The MIC range of 0.001–0.008% obtained during the establishment of the broth microdilution method [6] is covered by the MIC distribution observed during this study. It should be noted that the concentration of 0.008% was the highest concentration tested based on the solubility. The similarity of the results from the method development and the interlaboratory trial also shows that it is possible to use commercial microtiter plates in which chlorhexidine is present in dried form.

The RangeFinder software calculated a QC range of 0.001–0.016% (Table S5b). However, this range cannot be clearly defined, since the test range included in the microtiter plates is from 0.000008% to 0.008%. Therefore, a defined MIC of 0.016% cannot be determined since this would be ≥ 0.016%. This situation occurred in nine cases (two in lab 4, four in lab 5 and three in lab 6). An adaptation of the test range is also not possible due to the limited solubility of chlorhexidine in TSB medium in higher concentrations [5]. As mentioned before, QC ranges for AST should not contain more than three or four dilution steps [15]. However, there are QC ranges for AST with unspecified upper limits, which are available in the CLSI document VET01S [5]. Nevertheless, the inclusion of QC ranges with an unspecified upper limit does not allow proper quality control, e.g., in cases where the tested drug is not functional. Therefore, we decided not to set a QC range for *P. aeruginosa* ATCC^®^ 15442 and chlorhexidine. When testing *Pseudomonas* field isolates for their susceptibility to chlorhexidine, the MICs of *P. aeruginosa* ATCC^®^ 15442 can be compared with the MICs obtained in this study. However, for appropriate quality control of chlorhexidine, we recommend to use a different reference strain with an exactly defined QC range, such as the *E. coli* reference strain ATCC^®^ 10536.

#### 2.5.3. Polyhexanide

The polyhexanide MIC distribution for *P. aeruginosa* ATCC^®^ 15442 revealed at least six dilution steps ranging from 0.002% to ≥ 0.064%. The use of the media lots A, B and C resulted in at least four, at least six and three dilution steps. While lot B included all six dilution steps, lot A comprised the higher four and lot C the lower three dilution steps (Figure S4c).

At the laboratory level, seven laboratories (labs 1, 4, 5, 6, 7, 9, and 11) measured MICs of ≥0.064%. In total, 59 values (17.88%) were ≥ 0.064% and, therefore, could not clearly be defined as an exact MIC. In general, there was a wide distribution of the MIC values of at least four dilution steps (lab 8 and lab 10) up to at least six dilution steps (labs 4, 6, and 7) (Table S5c).

As already described for *P. aeruginosa* ATCC^®^ 15442 and chlorhexidine, there were MIC values above the highest test concentration. This information was not included in the evaluation using the RangeFinder software, which, therefore, suggested a QC range of 0.002–0.064% (Table S5c). This is not fitting our data set precisely. As mentioned above, there are QC ranges for AST without exact upper limits; however, this practice might weaken the power of the QC ranges. Therefore, we decided not to include this data set in the generation for QC ranges. As for chlorhexidine, the testing results of *P. aeruginosa* ATCC^®^ 15442 can be compared with the results of this study, but it is strongly recommended to include another reference strain for the QC of polyhexanide, with the suggestion to use *E. coli* ATCC^®^ 10536.

#### 2.5.4. Octenidine

The octenidine MIC distribution ranged from 0.000125% to 0.008% for *P. aeruginosa* ATCC^®^ 15442 and included seven dilution steps. Regarding the different media lots, only slight differences were seen. The MIC values obtained with lots A and C included four, and those obtained with lot B seven dilution steps (Figure S4d). Even though the MICs were widely distributed, most MIC values were within three dilution steps (0.00025–0.001%). At the laboratory level, the MIC distributions differed from three dilution steps (labs 1, 3, 7, 8, 9, and 10) up to seven dilution steps (lab 6). The results of lab 2 included six and those of the remaining laboratories four dilution steps (Table S5d).

The RangeFinder software determined a QC range of 0.000125–0.002% for *P. aeruginosa* ATCC^®^ 15442 and octenidine (Table S5d). This QC range includes five dilution steps and comprised 327 of the 330 MICs, representing 99.1% of all values.

#### 2.5.5. Summary *Pseudomonas aeruginosa* ATCC^®^ 15442

In comparison to the other reference strains, there were some problems regarding the testing of *P. aeruginosa* ATCC^®^ 15442 (Table S6). Due to the test ranges for chlorhexidine and polyhexanide, which mimic the solubility limits of the substances in TSB medium, the testing revealed MIC values in the upper range that could not be defined as an exact dilution step. Therefore, QC ranges for these two substances were not determined (see Section 2.5.2 and Section 2.5.3). The QC range for benzalkonium chloride comprised three dilution steps (0.002–0.008%) (see Section 2.5.1) and the octenidine QC range comprised five dilution steps (0.000125–0.008%) (see Section 2.5.4).

### 2.6. Overall Results and Outlook

The proposed QC ranges for the four reference strains selected as QC strains are summarized in Table 9. These newly determined QC ranges allow a validation of the BST of benzalkonium chloride, chlorhexidine, octenidine and polyhexanide using the respective reference strains in parallel testing with the corresponding test strains. QC ranges were established for all but two biocide–reference strain combinations. The proposed QC ranges for the biocides benzalkonium chloride and octenidine, comprised three or four dilution steps, whereas those for the two biguanides included four or five (chlorhexidine) or five (polyhexanide) dilution steps.

For polyhexanide, wide MIC distributions at the laboratory level with up to six and more dilution steps were seen for all reference strains. These findings might point towards a possible association of the observed wide ranges with the biocides tested. For chlorhexidine and octenidine, distributions of up to six or more dilution steps were only observed for *E. coli* ATCC^®^ 10536 and *P. aeruginosa* ATCC^®^ 15442, suggesting that the QC strain might also be a factor for these findings.

In total, 5280 MICs were measured by all the laboratories which participated in the interlaboratory trial. The QC ranges established for 14 of the 16 biocide–reference strain combinations are based on a total of 4620 MIC values. Among them, only 38 MICs (0.82%) were outside the proposed QC ranges. These MICs included all biocide–reference strain combinations, except chlorhexidine–*S. aureus* ATCC^®^ 6538, as well as benzalkonium chloride– and chlorhexidine–*E. hirae* ATCC^®^ 10541. At the biocide level, these deviant MICs were most frequently measured for octenidine (n = 14), followed by polyhexanide (n = 13), chlorhexidine (n = 6) and benzalkonium chloride (n = 5). However, for chlorhexidine and polyhexanide, only three QC ranges were included. At the reference strain level, the deviant MICs were detected most frequently for *E. coli* ATCC^®^ 10536 (n = 19), followed by *S. aureus* ATCC^®^ 6538 (n = 10), *E. hirae* ATCC^®^ 10541 (n = 5) and *P. aeruginosa* ATCC^®^ 15442 (n = 4). Here, it should be noted that for the latter QC strain, only two QC ranges were included in the evaluation. These findings are in agreement with the fact that the largest number of deviating MICs (n = 7) was detected for the combination octenidine–*E. coli* ATCC^®^ 10536. At the laboratory level, all MICs determined by labs 2, 8, 9 and 10 were within the proposed QC ranges, whereas one to four deviating MICs were determined by labs 1, 3–7. Only lab 11 produced more than half of all MICs outside the proposed QC ranges (n = 20). Differences in the results determined by the same laboratory might be due to individual factors, such as different persons performing the tests and reading the MIC values during the ten repetitions. This observation underlined the necessity to have QC ranges for BST as they function as internal controls of the test system and enable determining whether the test results can be considered as valid or whether the test has to be repeated. Bearing in mind that each of the eleven participating laboratories provided 480 MICs, with 420 being included in the development of the 14 QC ranges, even 20 of 420 MICs outside the proposed QC range accounted for only 4.76% of all MICs included in the calculation of the QC range and provided by this laboratory. Thus, the percentages of MIC values within the determined QC ranges per laboratory varied between 95.24 and 100%, which confirmed that the BST method is highly stable and very similar results can be obtained in different laboratories when testing the same bacterial strains under defined conditions.

The importance of biocide susceptibility testing might increase in the future, since decreased susceptibility to biocides has already been determined. For example, decreased susceptibility to benzalkonium chloride has been described for *Pseudomonas* spp. from poultry carcasses [16], low levels of reduced susceptibility to quaternary ammonium compounds among *Listeria monocytogenes*, *Staphylococcus* spp., *Pseudomonas* spp., lactic acid bacteria and coliforms from food and food processing industry were detected [17] and reduced susceptibility to triclosan in *P. aeruginosa* was found in a heavily contaminated triclosan soap dispenser in a hospital [18].

The recently developed BST has already been used in some studies to test *S. aureus* from horses [19], primates [20], and beavers [21] as well as *E. coli* from animal origin [22,23]. It should be noted that conventional biocides, such as the ones described during this study, have a toxic potential. Therefore, it is very important to find more safe, eco-friendly and effective alternative biocide products [24]. Further studies are needed to allow proper testing (including QC) of such green biocides in the future.

## 3. Materials and Methods

### 3.1. Selection of the Bacterial Strains and Biocides

Four reference strains, namely *S. aureus* ATCC^®^ 6538, *E. hirae* ATCC^®^ 10541, *E. coli* ATCC^®^ 10536 and *P. aeruginosa* ATCC^®^ 15442, were selected for the development of QC ranges. These reference strains have been previously used for the development of a broth macrodilution method [5] and a broth microdilution method [6], and are commonly used for BET [3,4]. Four biocides were used, including a quaternary ammonium compound (benzalkonium chloride; test range: 0.000008–0.016%), two biguanides (chlorhexidine; test range: 0.000008–0.008%, and polyhexanide; test range: 0.000015–0.032%) and a bispyridine (octenidine; test range: 0.000015–0.016%) in commercially produced microtiter plates (sifin diagnostics GmbH, Berlin, Germany) (Figure S5).

### 3.2. Interlaboratory Trial

An interlaboratory trial including eleven laboratories was conducted for the development of QC ranges for BST from 5 October to 15 December 2020. The participants comprised four federal research laboratories, five university diagnostic laboratories, one regional state laboratory and one private veterinary service laboratory. All laboratories performed the testing successfully for all reference strain–biocide combinations according to the protocol, without encountering major problems. However, there were some difficulties in reading the results due to little growth after 24 h or precipitations in the wells. In all laboratories, the bacterial cell counts were determined in parallel to the tests and, for inclusion of data sets, the values had to be within the acceptable range of 1 × 10^8^–1 × 10^9^ CFU/mL [5,6,14]. This prerequisite was an inclusion criteria to assure the quality of the data set.

Our protocol was based on the recommendations for the development of QC ranges for AST by the CLSI document VET02-A3 [15] with some modifications. Each laboratory tested each reference strain ten times using one lot of commercially produced microtiter plates and in parallel three lots of tryptic soy broth (TSB) (Tryptic Soy Broth Merck, lot VM884959921, Darmstadt, Germany; CASO-Bouillon Roth, lot 300296352, Karlsruhe, Germany; CASO-Bouillon (TSB) Th. Geyer, lot 180122, Stuttgart, Germany). TSB was purchased as powder and aliquots were distributed to the participating laboratories, where the media were prepared according to the manufacturers’ instructions. Since there are no approved QC ranges for BST available, it was not possible to include biocides with existing QC ranges in this study. The tests were performed within at least three days, with no more than four tests per reference strain and media lot per day. The test was performed according to the protocol by Schug et al. [6] with some modifications. Since custom-made microtiter plates with dry biocides were used, the inoculum density had to be adjusted to 25 µL per 10 mL single concentrated TSB to reach corresponding bacterial densities in the wells as in previous studies [5,6]. Then—for each test—half a microtiter plate (lanes A–D or lanes E–H; Figure S5) was inoculated with 100 µL inoculum per well. After finishing the experiments, the results were recorded in the provided Excel files and subjected to comparative evaluations.

### 3.3. Data Analysis

The tests of all eleven laboratories resulted in 330 data points for each biocide–reference strain combination. The laboratories were anonymized and coded as lab 1 to lab 11. The media lots were coded as lot A to lot C. Data analysis was conducted using the RangeFinder software, a statistical method described by Turnidge and Bordash [25]. For this, the MIC values obtained for each biocide–reference strain combination were entered for each biocide for the different reference strains. For each laboratory, the MICs were entered per media lot in the “data entry” of the software. The software then calculated the respective QC ranges. However, since the MIC values of the biocides are given in % and the software was generated to process MIC values in mg/L and different dilution steps, a transformation of % into mg/L was necessary to perform the calculation via RangeFinder. The calculated values were rounded up to the next higher dilution step.

## 4. Conclusions

BST is a helpful tool to monitor the development of changes in biocide susceptibility of bacterial pathogens in human and veterinary medicine, agriculture, food production and other areas with high selection pressure due to the use of biocides. The correct performance of laboratory tests, including BST, requires adequate quality controls. However, neither QC strains nor QC ranges for specific biocides have been available so far. In this study, QC ranges were determined for the first time for the four widely used biocides benzalkonium chloride, chlorhexidine, polyhexanide, and octenidine applicable to the reference strains *S. aureus* ATCC^®^ 6538, *E. hirae* ATCC^®^ 10541, and *E. coli* ATCC^®^ 10536. For *P. aeruginosa* ATCC^®^ 15442, only QC ranges for benzalkonium chloride and octenidine could be determined. These novel QC strains and their defined QC ranges represent an important addition to the BST methodology as they represent the first internal controls to validate the results obtained by BST via broth microdilution. QC ranges for further biocides, including green biocides, need to be determined to allow validated BST of additional biocides in the future. The ability to use commercially manufactured microtiter plates for BST will harmonize and simplify BST.

## Figures and Tables

**Table 1:** Interlaboratory comparison of the MICs measured when testing *S. aureus* ATCC^®^ 6538 with benzalkonium chloride.

MIC (in %)	Lab 1	Lab 2	Lab 3	Lab 4	Lab 5	Lab 6	Lab 7	Lab 8	Lab 9	Lab 10	Lab 11
≥0.032											
0.016											
0.008											
0.004											
0.002											
0.001					*3*						
0.0005											
0.00025				3					1		
0.000125	17	10	11	15		9	2	30	7	7	19
0.00006	13	20	19	12	27	20	28		22	23	11
0.00003						1					
0.000015											
≤0.000008											

The white area represents the proposed QC range. The gray areas represent the parts of the test range outside the QC range. Values outside this QC range are displayed in red and italics.

**Table 2:** Interlaboratory comparison of the MICs measured when testing *S. aureus* ATCC^®^ 6538 with chlorhexidine.

MIC (in %)	Lab 1	Lab 2	Lab 3	Lab 4	Lab 5	Lab 6	Lab 7	Lab 8	Lab 9	Lab 10	Lab 11
≥0.016											
0.008											
0.004											
0.002											
0.001											
0.0005											
0.00025				3							
0.000125	20	11	17	20	6	5		8	4	8	27
0.00006	10	18	13	7	22	25	28	22	26	22	3
0.00003		1			2		2				
0.000015											
≤0.000008											

The white area represents the proposed QC range. The gray areas represent the parts of the test range outside the QC range.

**Table 3:** Interlaboratory comparison of the MICs measured when testing *S. aureus* ATCC^®^ 6538 with polyhexanide.

MIC (in %)	Lab 1	Lab 2	Lab 3	Lab 4	Lab 5	Lab 6	Lab 7	Lab 8	Lab 9	Lab 10	Lab 11
≥0.064											
0.032											
0.016											
0.008											
0.004											
0.002						*2*					*3*
0.001				3				2			13
0.0005	4	2		5				6	2	7	12
0.00025	6	11	10	15	6	3	6	9	8	14	2
0.000125	20	15	20	7	16	24	24	13	19	9	
0.00006		2			8	1			1		
0.00003											
≤0.000015											

The white area represents the proposed QC range. The gray areas represent the parts of the test range outside the QC range. Values outside this QC range are displayed in red and italics.

**Table 4:** Interlaboratory comparison of the MICs measured when testing *S. aureus* ATCC^®^ 6538 with octenidine.

MIC (in %)	Lab 1	Lab 2	Lab 3	Lab 4	Lab 5	Lab 6	Lab 7	Lab 8	Lab 9	Lab 10	Lab 11
≥0.032											
0.016											
0.008											
0.004											
0.002											
0.001											
0.0005	*1*										
0.00025	14	8	15	14	5	2	1	8	1	3	15
0.000125	14	18	14	14	21	24	9	17	23	25	15
0.00006	1	4	1	2	4	3	20	5	6	2	
0.00003						*1*					
≤0.000015											

The white area represents the proposed QC range. The gray areas represent the parts of the test range outside the QC range. Values outside this QC range are displayed in red and italics.

**Table 5:** Interlaboratory comparison of the MICs measured when testing *E. coli* ATCC^®^ 10536 with benzalkonium chloride.

MIC (in %)	Lab 1	Lab 2	Lab 3	Lab 4	Lab 5	Lab 6	Lab 7	Lab 8	Lab 9	Lab 10	Lab 11
≥0.032											
0.016											
0.008											
0.004											
0.002	2		4	10				2	6		1
0.001	21	25	19	13	18	15	12	28	21	26	19
0.0005	7	5	7	7	12	14	18		3	4	10
0.00025						*1*					
0.000125											
0.00006											
0.00003											
0.000015											
≤0.000008											

The white area represents the proposed QC range. The gray areas represent the parts of the test range outside the QC range. Values outside this QC range are displayed in red and italics.

**Table 6:** Interlaboratory comparison of the MICs measured when testing *E. coli* ATCC^®^ 10536 with chlorhexidine.

MIC (in %)	Lab 1	Lab 2	Lab 3	Lab 4	Lab 5	Lab 6	Lab 7	Lab 8	Lab 9	Lab 10	Lab 11
≥0.016											
0.008											
0.004											
0.002											
0.001											*1*
0.0005			*1*								*4*
0.00025	5	2	5	13				3		4	15
0.000125	11	5	5	10	5	1		2	2	4	7
0.00006	14	16	14	6	12	9	5	25	17	21	2
0.00003		7	5	1	13	20	25		11	1	1
0.000015											
≤0.000008											

The white area represents the proposed QC range. The gray areas represent the parts of the test range outside the QC range. Values outside this QC range are displayed in red and italics.

**Table 7:** Interlaboratory comparison of the MICs measured when testing *E. coli* ATCC^®^ 10536 with polyhexanide.

MIC (in %)	Lab 1	Lab 2	Lab 3	Lab 4	Lab 5	Lab 6	Lab 7	Lab 8	Lab 9	Lab 10	Lab 11
≥0.064											*1*
0.032											
0.016											
0.008											
0.004											
0.002											*4*
0.001			1	1							5
0.0005	2	2	4	12				1	3	6	11
0.00025	11	18	18	12	9	1	7	7	12	10	8
0.000125	17	10	7	5	16	29	21	22	15	14	1
0.00006					5		2				
0.00003											
≤0.000015											

The white area represents the proposed QC range. The gray areas represent the parts of the test range outside the QC range. Values outside this QC range are displayed in red and italics.

**Table 8:** Interlaboratory comparison of the MICs measured when testing *E. coli* ATCC^®^ 10536 with octenidine.

MIC (in %)	Lab 1	Lab 2	Lab 3	Lab 4	Lab 5	Lab 6	Lab 7	Lab 8	Lab 9	Lab 10	Lab 11
≥0.032											
0.016											
0.008											
0.004											
0.002											*1*
0.001			*1*	*2*							*3*
0.0005	2	2	5	12	1			2		2	9
0.00025	20	15	18	8	15	2		10	2	15	13
0.000125	8	13	6	8	14	16	20	15	26	12	3
0.00006						12	10	3	2	1	1
0.00003											
≤0.000015											

The white area represents the proposed QC range. The gray areas represent the parts of the test range outside the QC range. Values outside this QC range are displayed in red and italics.

**Table 9:** Summary of the QC ranges.

Biocide	QC Strains			
	*Staphylococcus aureus* ATCC^®^ 6538 (DSM 799)	*Enterococcus hirae* ATCC^®^ 10541 (DSM 3320)	*Escherichia coli* ATCC^®^ 10536 (DSM 682)	*Pseudomonas aeruginosa* ATCC^®^ 15442 (DSM 939).
**benzalkonium chloride**	0.00003–0.00025%	0.000125–0.0005%	0.0005–0.002%	0.002–0.008%
**chlorhexidine**	0.00003–0.00025%	0.00003–0.00025%	0.000015–0.00025%	-
**polyhexanide**	0.00006–0.001%	0.000125–0.002%	0.00006–0.001%	-
**octenidine**	0.00006–0.00025%	0.00006–0.0005%	0.00006–0.0005%	0.000125–0.002%

## Data Availability

All relevant data used in the manuscript is available within the manuscript and the Supplemental files.

## Supplementary Materials

The following are available online at https://www.mdpi.com/article/10.3390/pathogens11020223/s1, Figure S1: Differences of the media lots for *S. aureus* ATCC^®^ 6538; Figure S2: Differences of the media lots for *E. hirae* ATCC^®^ 10541; Figure S3: Differences of the media lots for *E. coli* ATCC^®^ 10536, Figure S4: Differences of the media lots for *P. aeruginosa* ATCC^®^ 15442; Figure S5: Layout of the microtiter plate “MICRONAUT-S Biozid MHK FU-Berlin”; Table S1: Summary of the QC ranges of *S. aureus* ATCC^®^ 6538; Table S2: QC ranges for *E. hirae* ATCC^®^ 10541; Table S3: Summary of the QC ranges of *E. hirae* ATCC^®^ 10541; Table S4: Summary of the QC ranges of *E. coli* ATCC^®^ 10536; Table S5: QC ranges for *P. aeruginosa* ATCC^®^ 15442; Table S6: Summary of the QC ranges of *P. aeruginosa* ATCC^®^ 15442.
