# Supplementary material for: Development of Quality Control Ranges for Biocide Susceptibility Testing
Source: Pathogens. 2022 Feb 8;11(2):223. doi: 10.3390/pathogens11020223 (PMC8878709; doi:10.3390/pathogens11020223)
Supplement: Supplementary file 1 [file pathogens-11-00223-s001.zip › pathogens-1547182-supplementary/Figure S1 S. aureus-color.pdf]

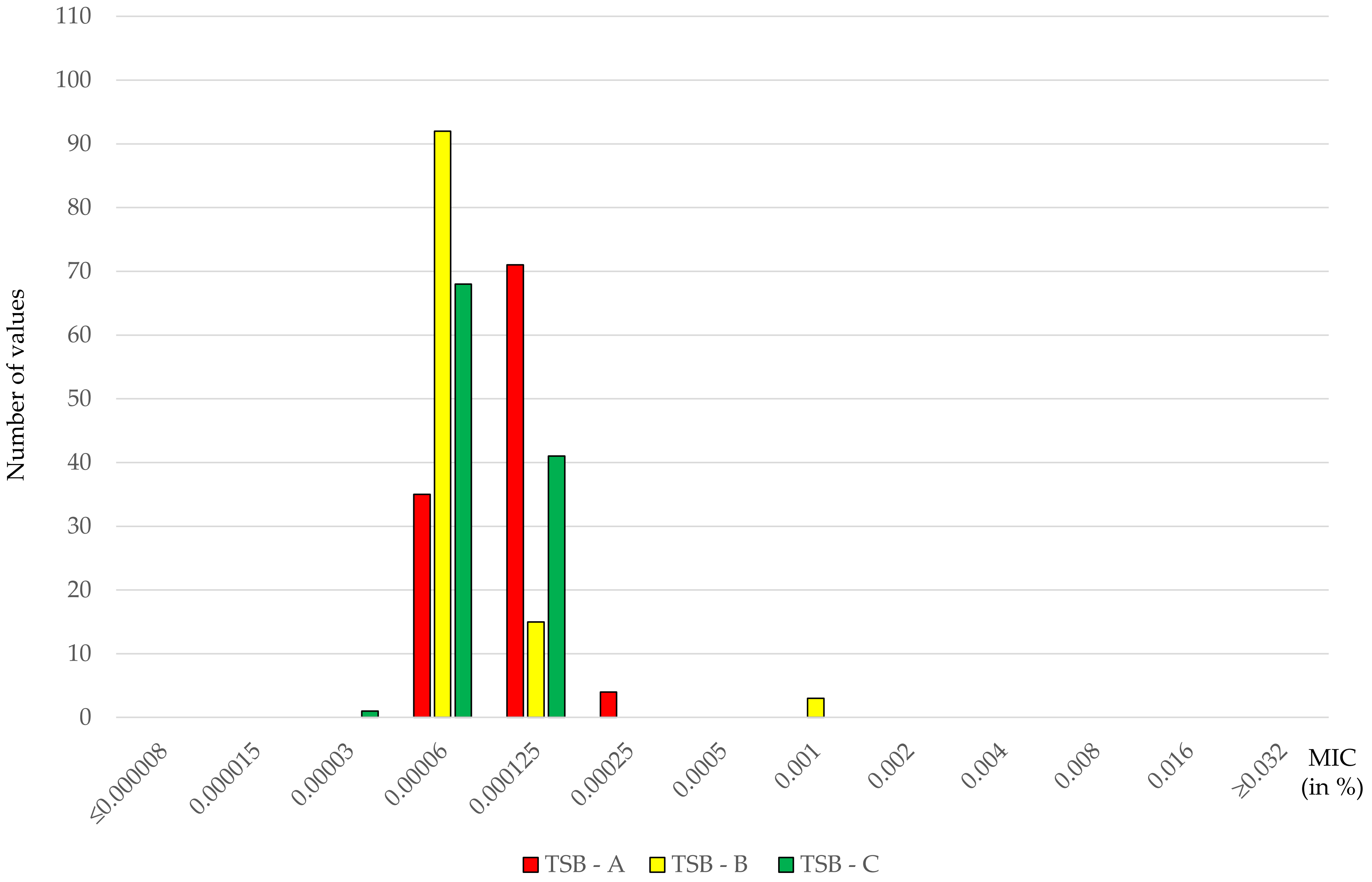

Figure S1a: Differences of the media lots for *S. aureus* ATCC® 6538 and benzalkonium chloride

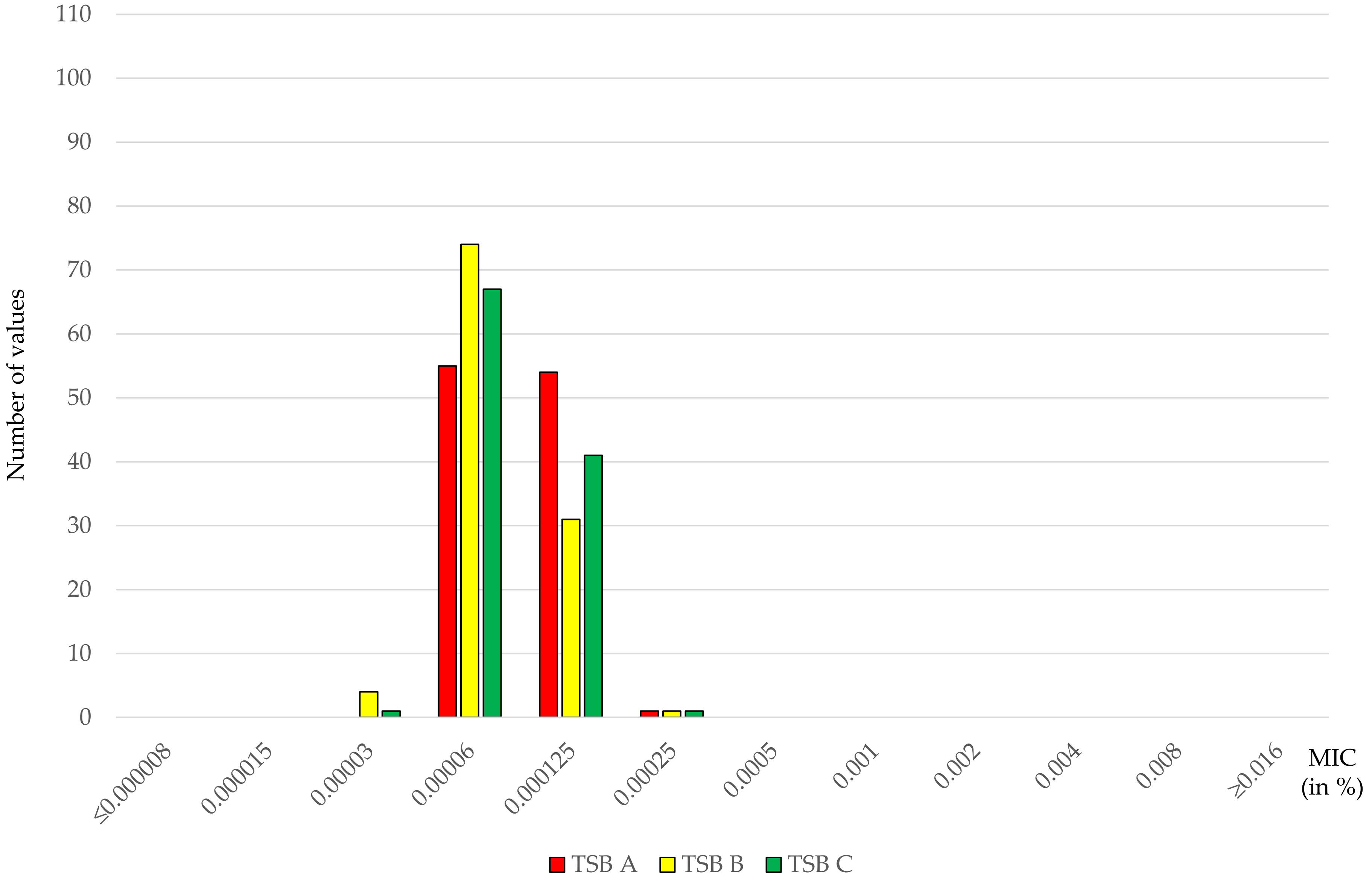

Figure S1b: Differences of the media lots for *S. aureus* ATCC® 6538 and chlorhexidine

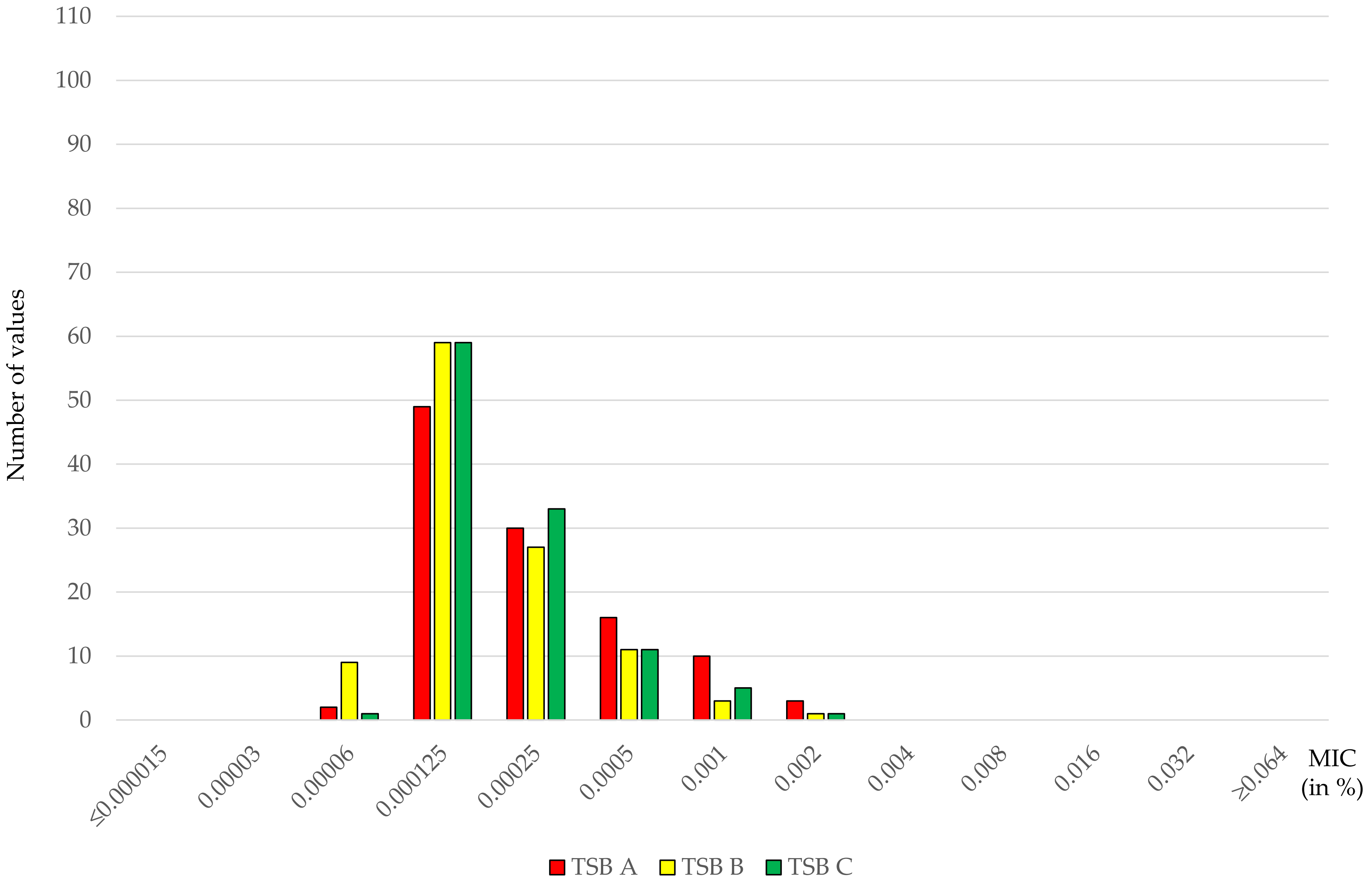

Figure S1c: Differences of the media lots for *S. aureus* ATCC® 6538 and polyhexanide

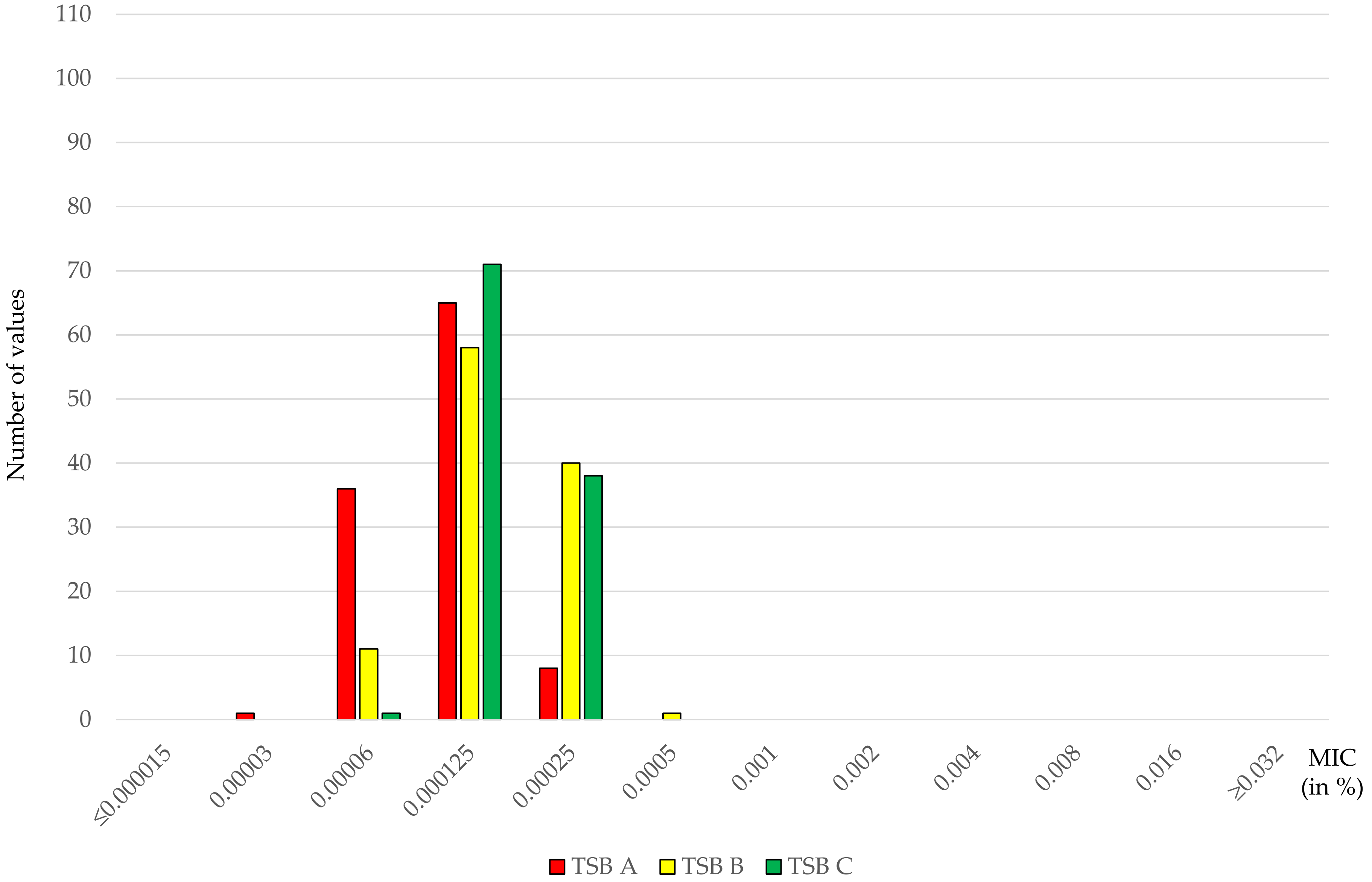

Figure S1d: Differences of the media lots for *S. aureus* ATCC® 6538 and octenidine
