# Supplementary material for: Development of Quality Control Ranges for Biocide Susceptibility Testing
Source: Pathogens. 2022 Feb 8;11(2):223. doi: 10.3390/pathogens11020223 (PMC8878709; doi:10.3390/pathogens11020223)
Supplement: Supplementary file 1 [file pathogens-11-00223-s001.zip › pathogens-1547182-supplementary/Figure S2 E. hirae-color.pdf]

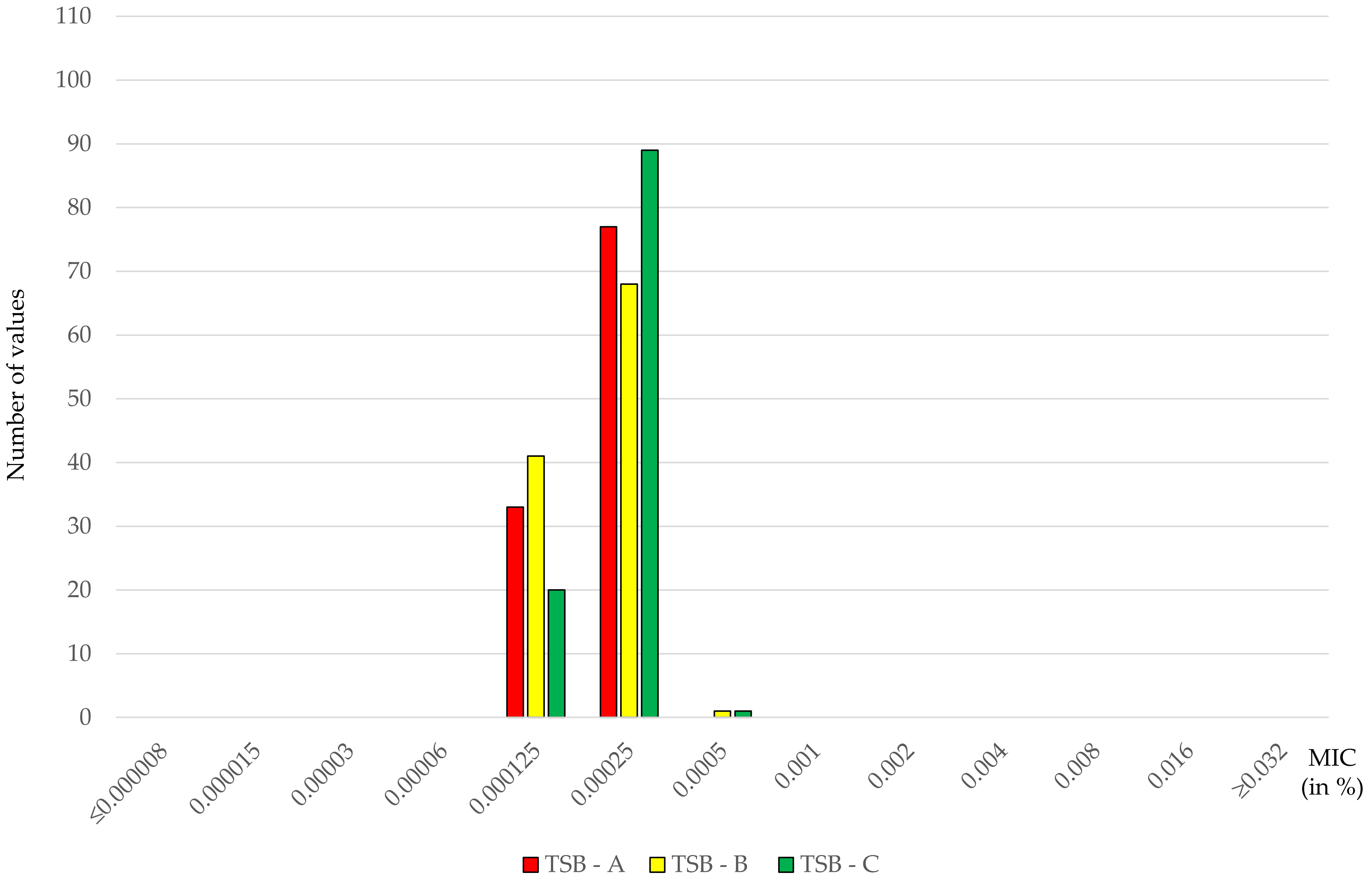


Figure S2a: Differences of the media lots for *E. hirae* ATCC® 10541 and benzalkonium chloride

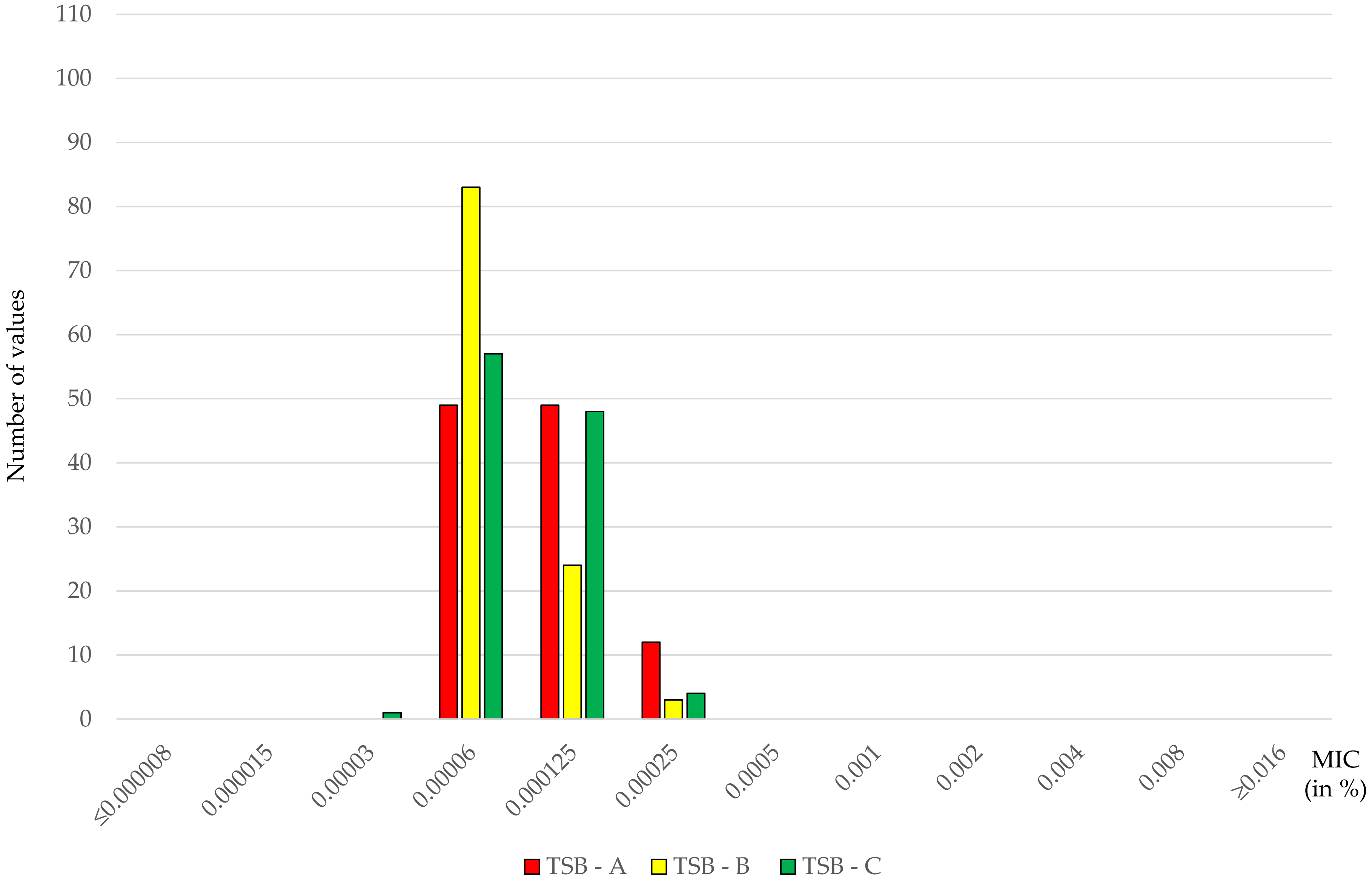


Figure S2b: Differences of the media lots for *E. hirae* ATCC® 10541 and chlorhexidine

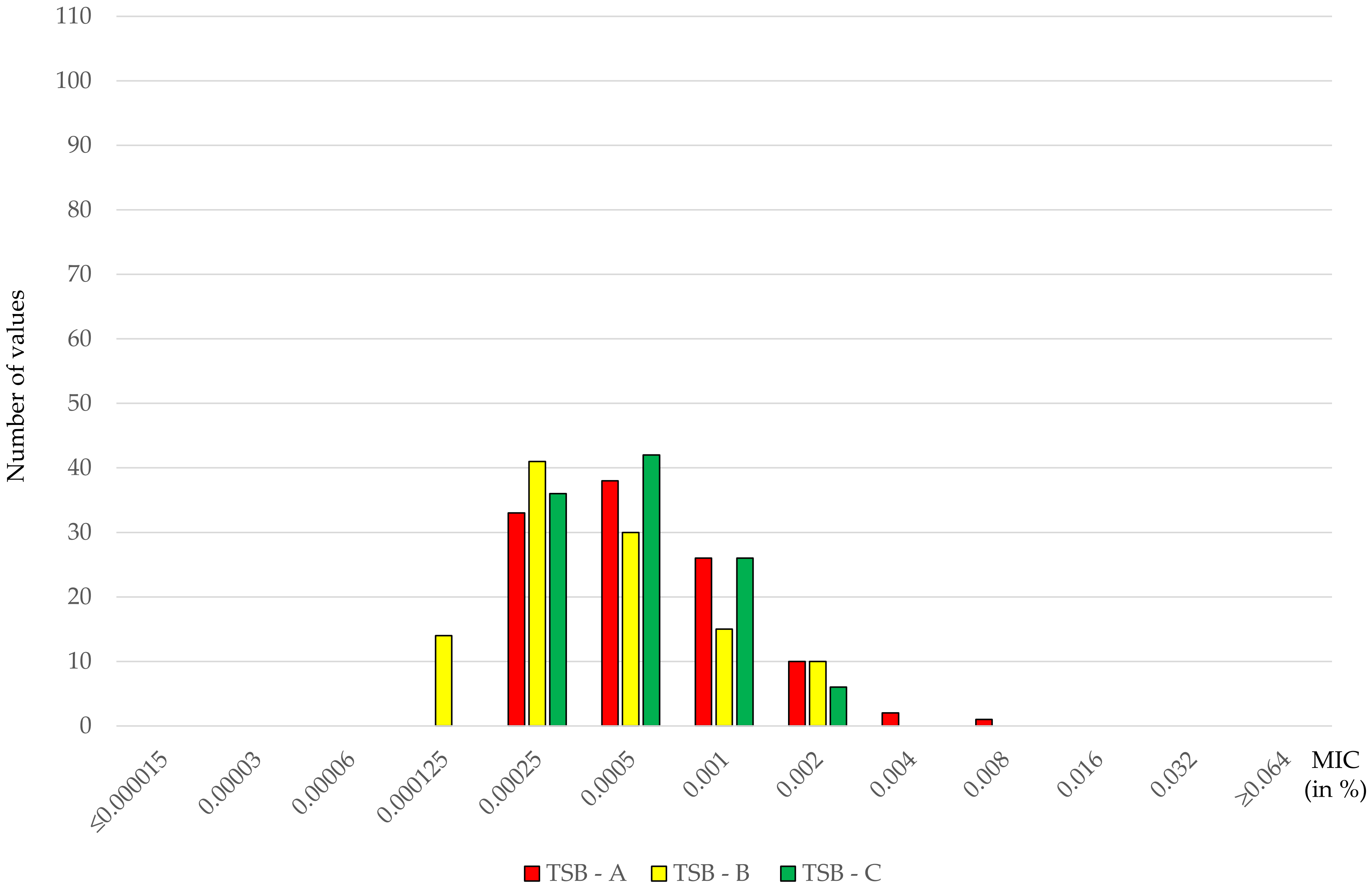


Figure S2c: Differences of the media lots for *E. hirae* ATCC® 10541 and polyhexanide

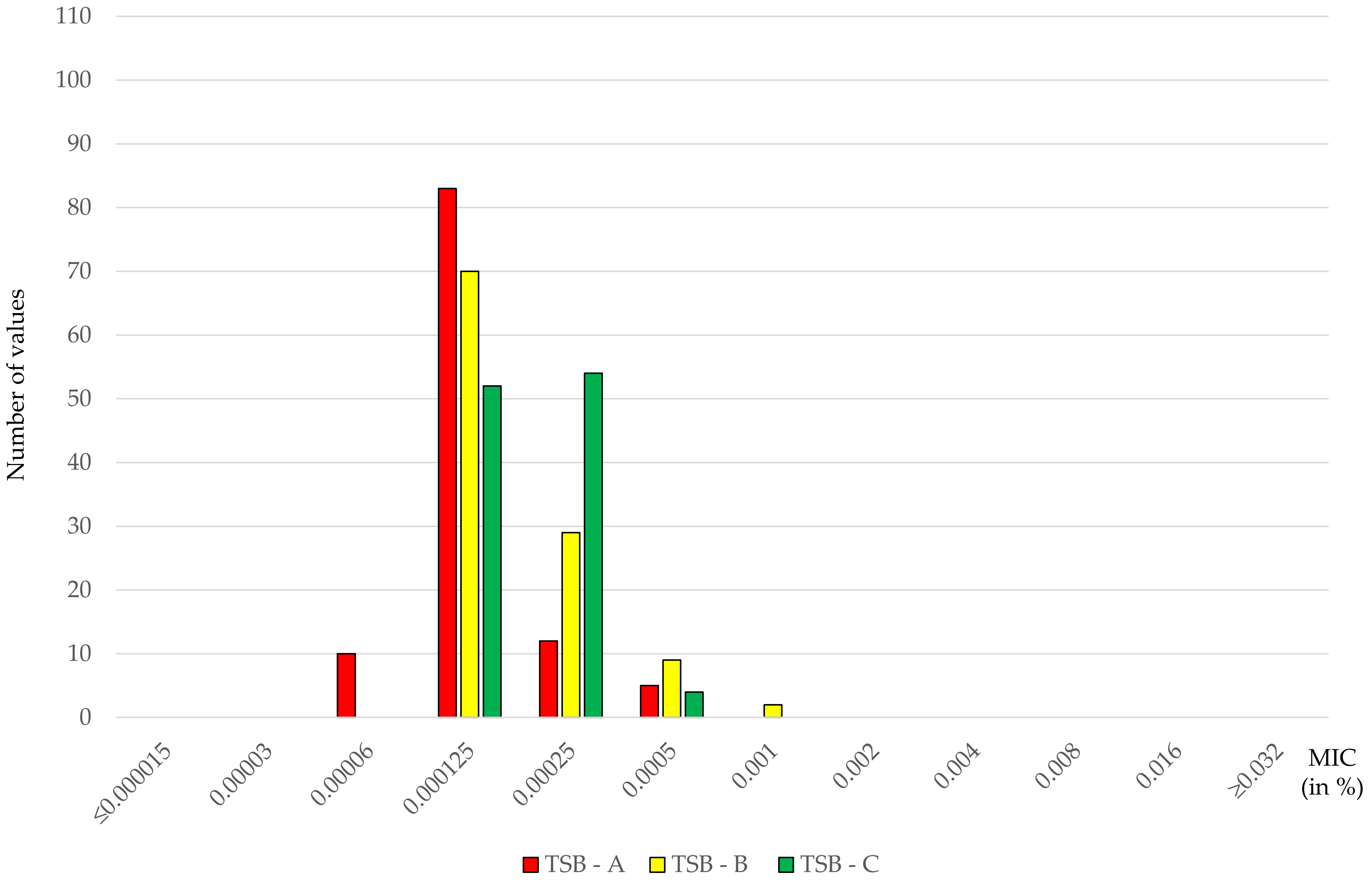


Figure S2d: Differences of the media lots for *E. hirae* ATCC® 10541 and octenidine
