# Supplementary material for: Development of Quality Control Ranges for Biocide Susceptibility Testing
Source: Pathogens. 2022 Feb 8;11(2):223. doi: 10.3390/pathogens11020223 (PMC8878709; doi:10.3390/pathogens11020223)
Supplement: Supplementary file 1 [file pathogens-11-00223-s001.zip › pathogens-1547182-supplementary/Figure S3 E. coli-color.pdf]

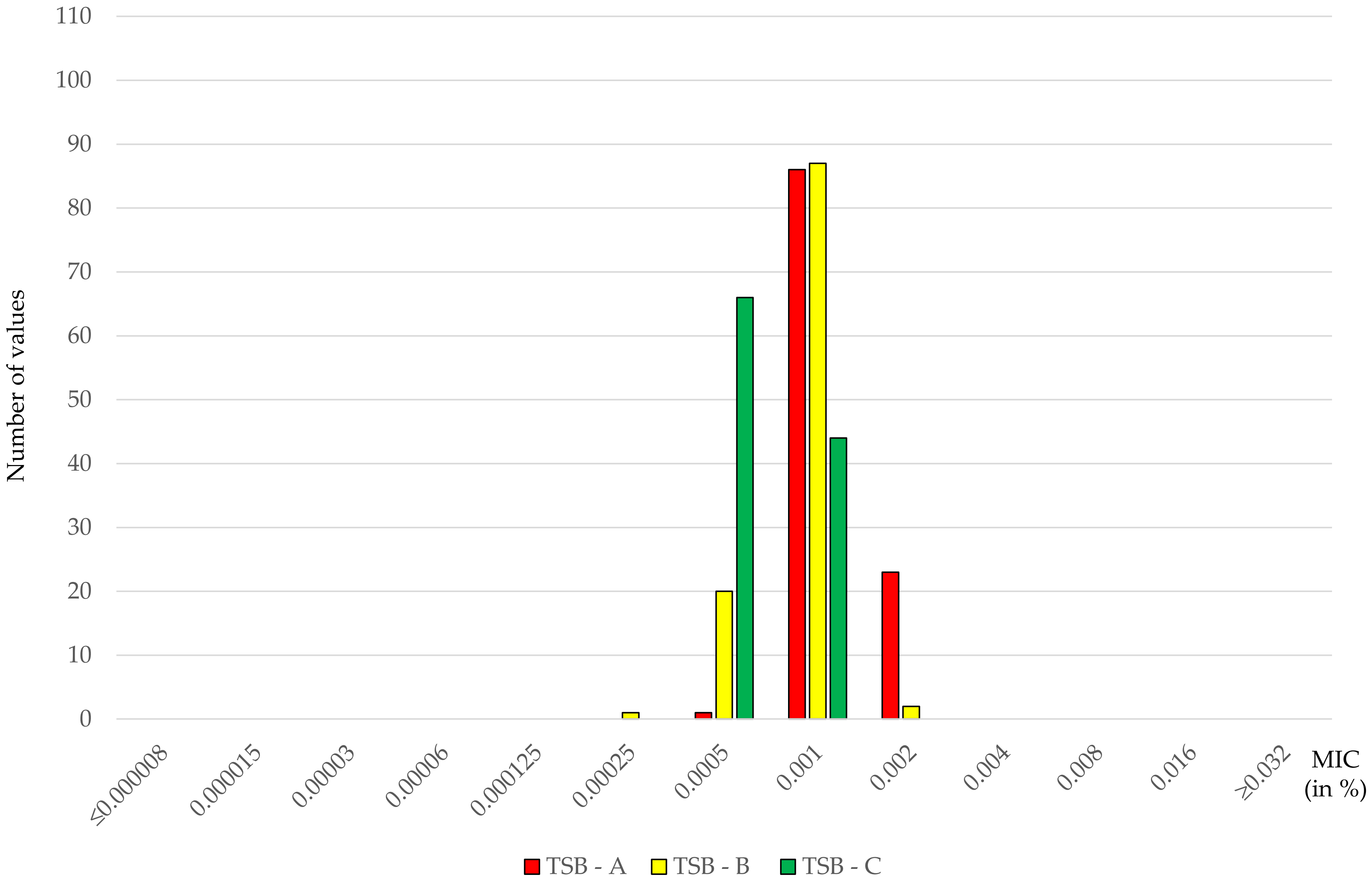

Figure S3a: Differences of the media lots for *E. coli* ATCC® 10536 and benzalkonium chloride

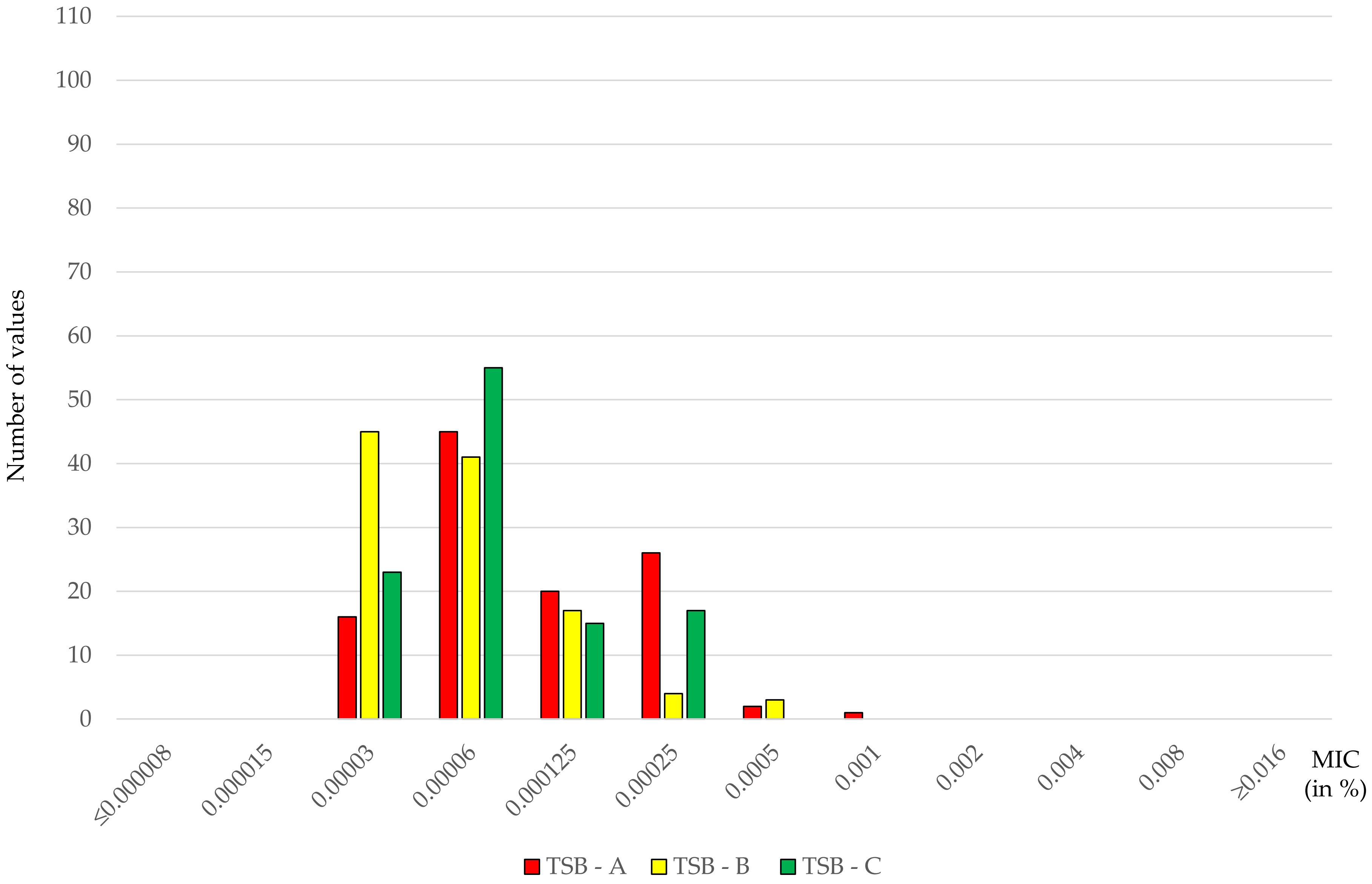

Figure S3b: Differences of the media lots for *E. coli* ATCC® 10536 and chlorhexidine

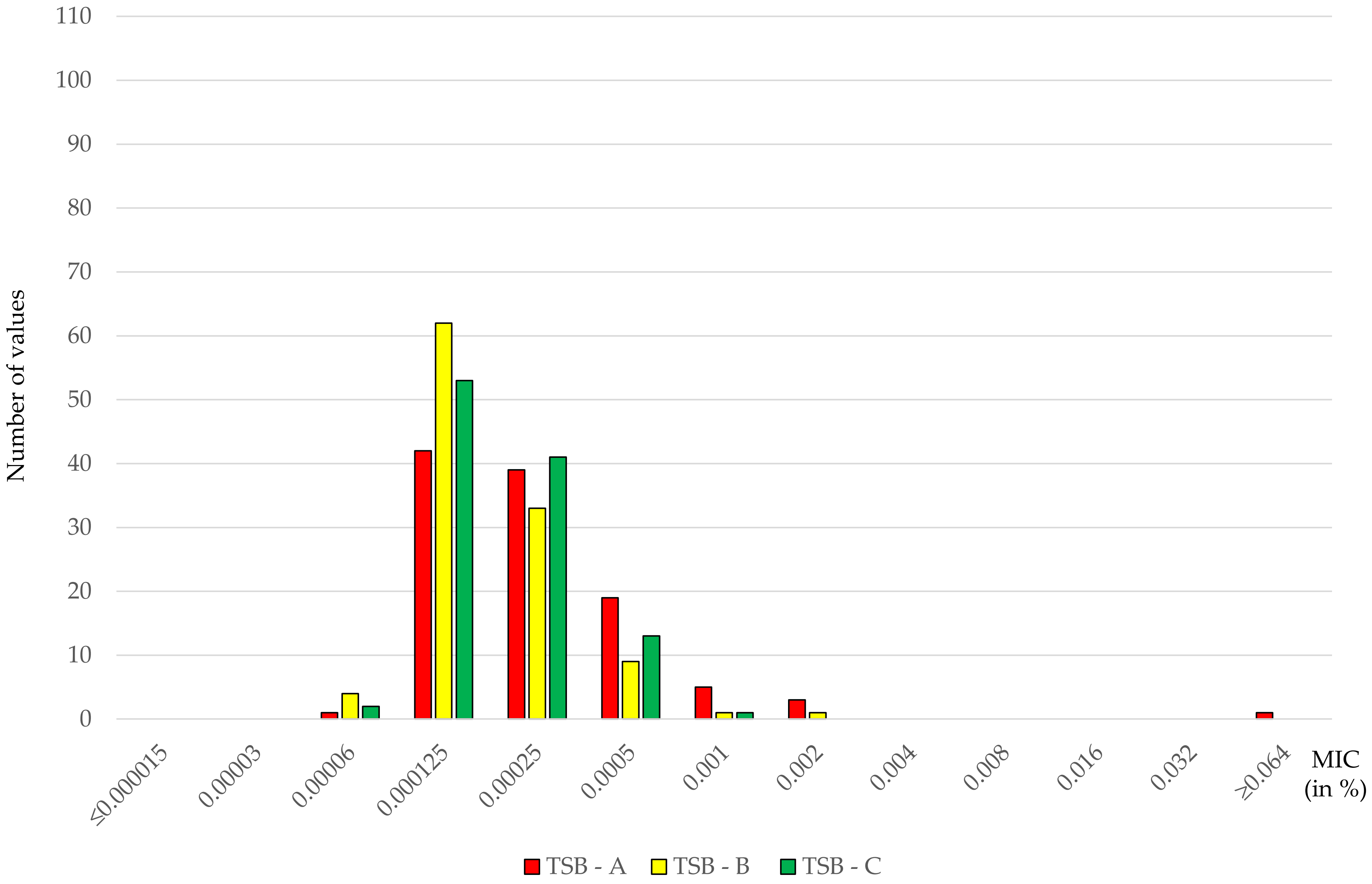

Figure S3c: Differences of the media lots for *E. coli* ATCC® 10536 and polyhexanide

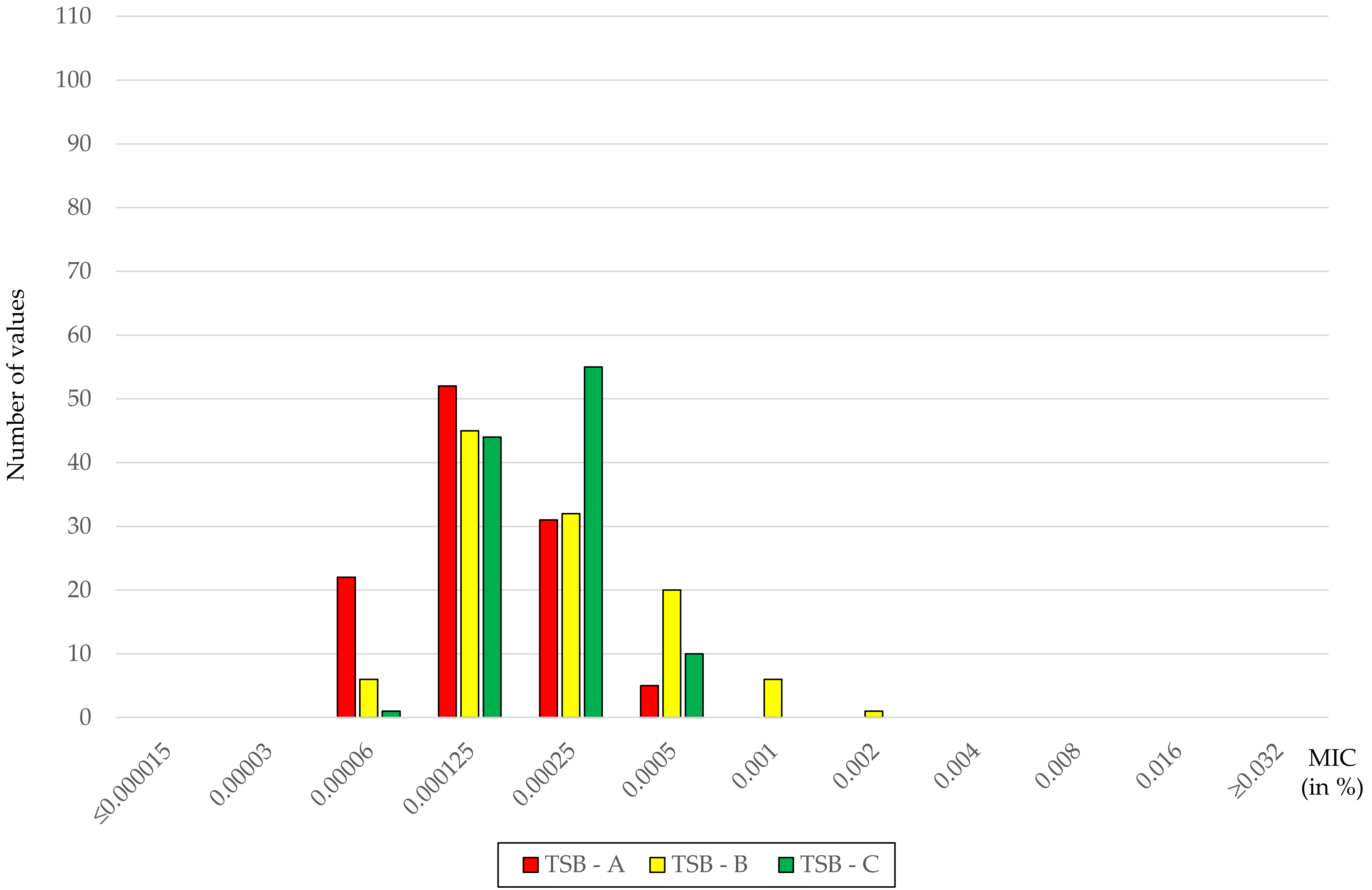

Figure S3d: Differences of the media lots for *E. coli* ATCC® 10536 and octenidine
