# Supplementary material for: Development of Quality Control Ranges for Biocide Susceptibility Testing
Source: Pathogens. 2022 Feb 8;11(2):223. doi: 10.3390/pathogens11020223 (PMC8878709; doi:10.3390/pathogens11020223)
Supplement: Supplementary file 1 [file pathogens-11-00223-s001.zip › pathogens-1547182-supplementary/Figure S4 P. aeruginosa-color.pdf]

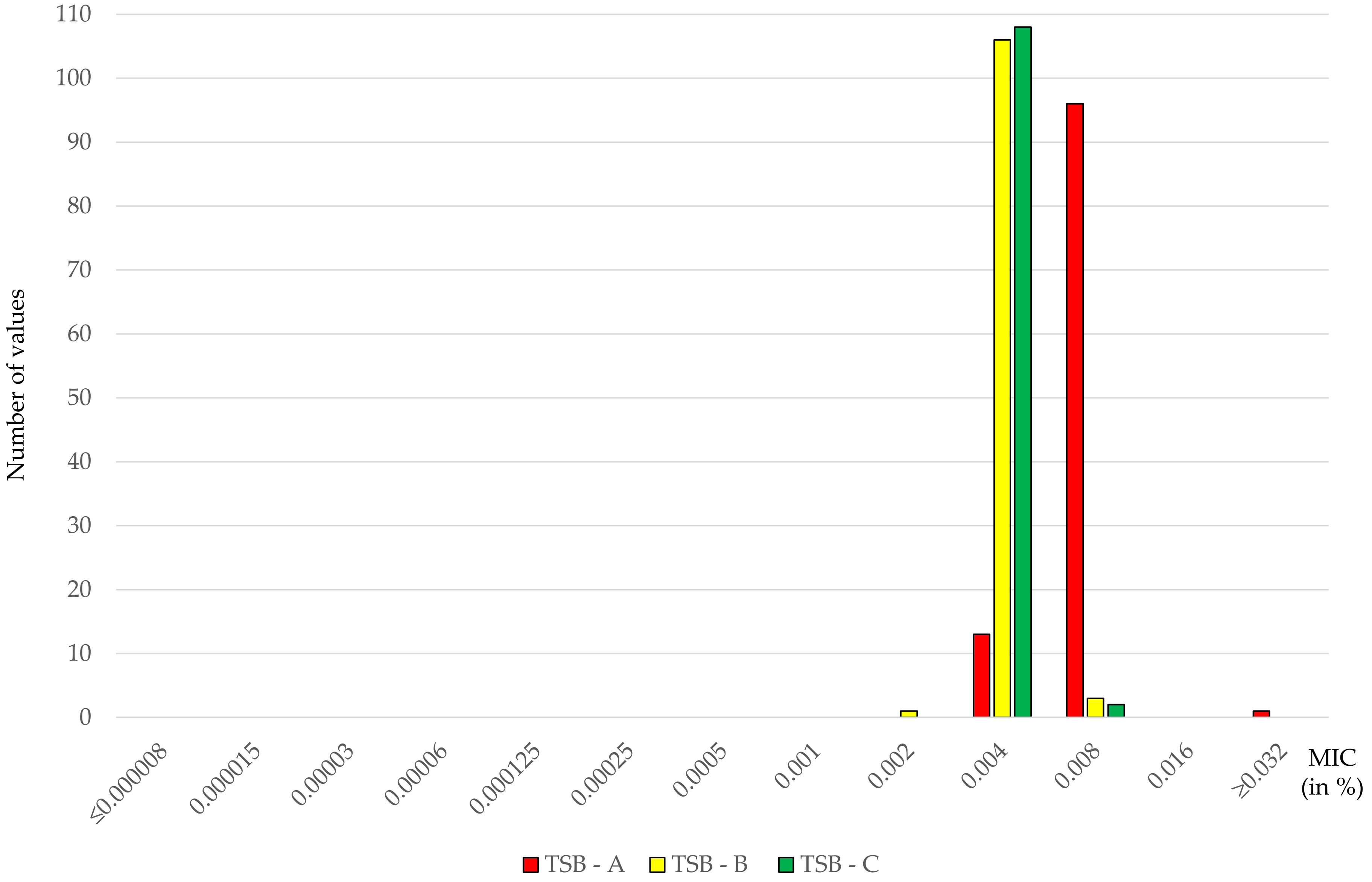

Figure S4a: Differences of the media lots for *P. aeruginosa* ATCC® 15442 and benzalkonium chloride

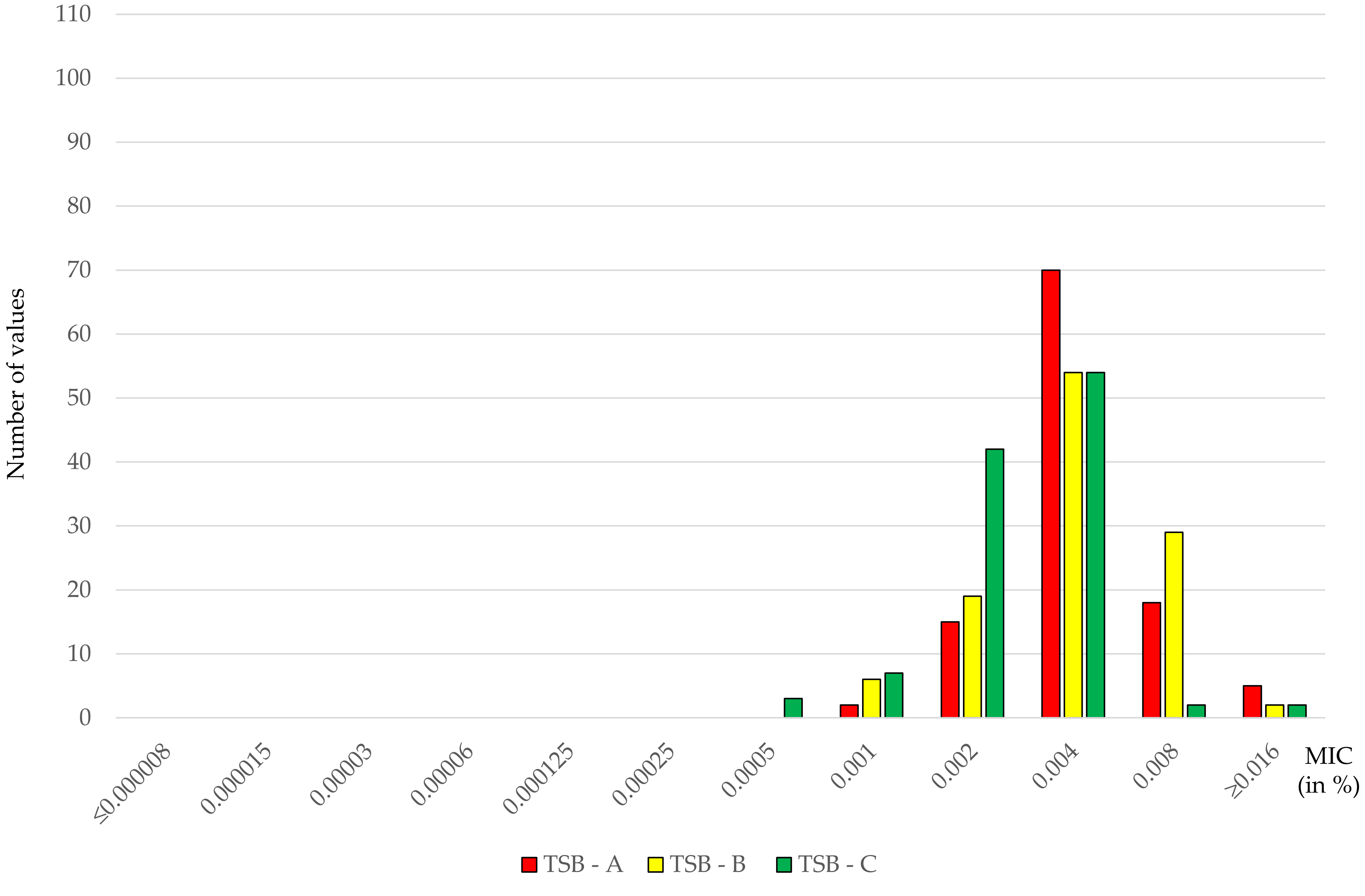

Figure S4b: Differences of the media lots for *P. aeruginosa* ATCC® 15442 and chlorhexidine

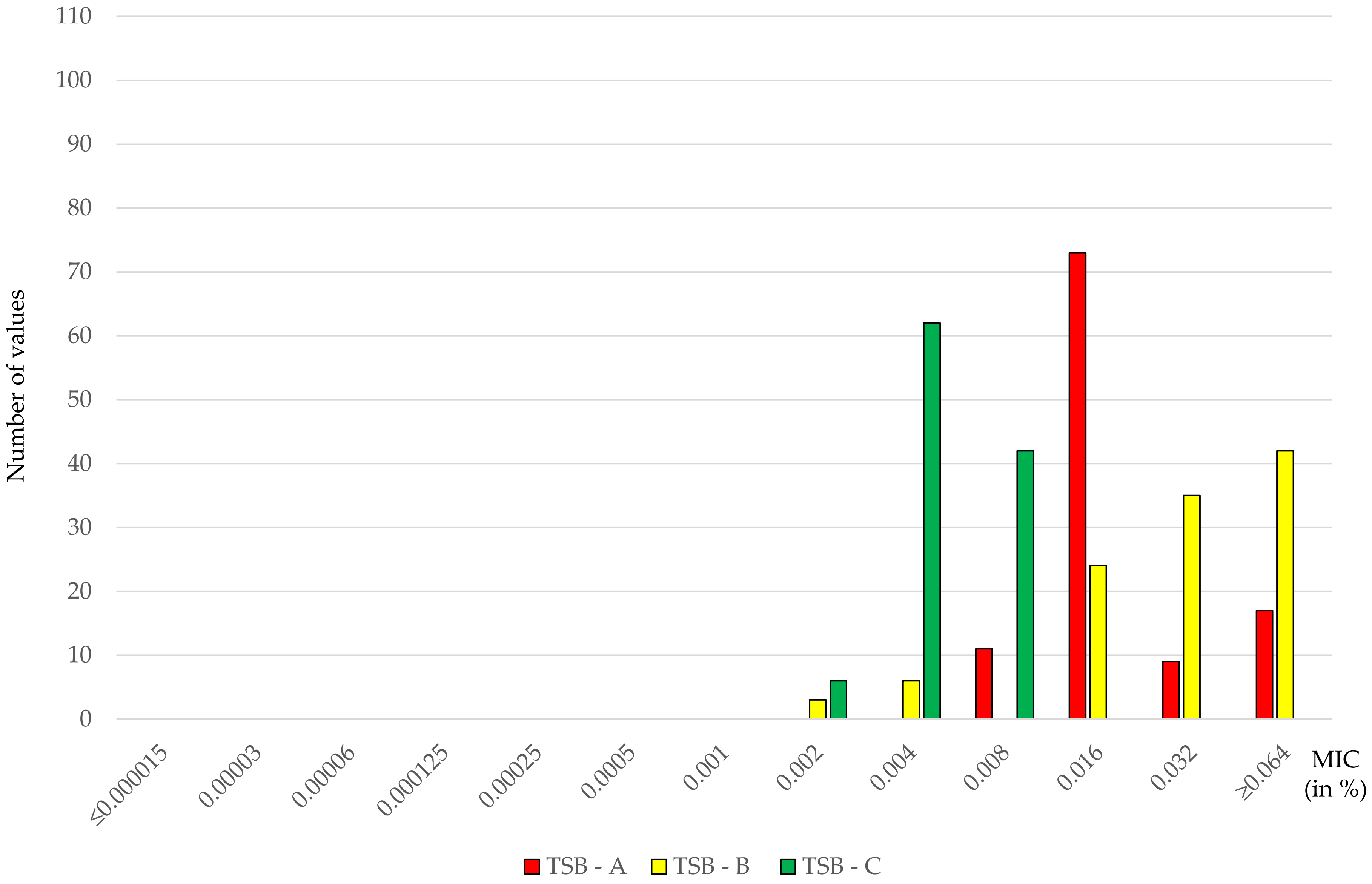

Figure S4c: Differences of the media lots for *P. aeruginosa* ATCC® 15442 and polyhexanide

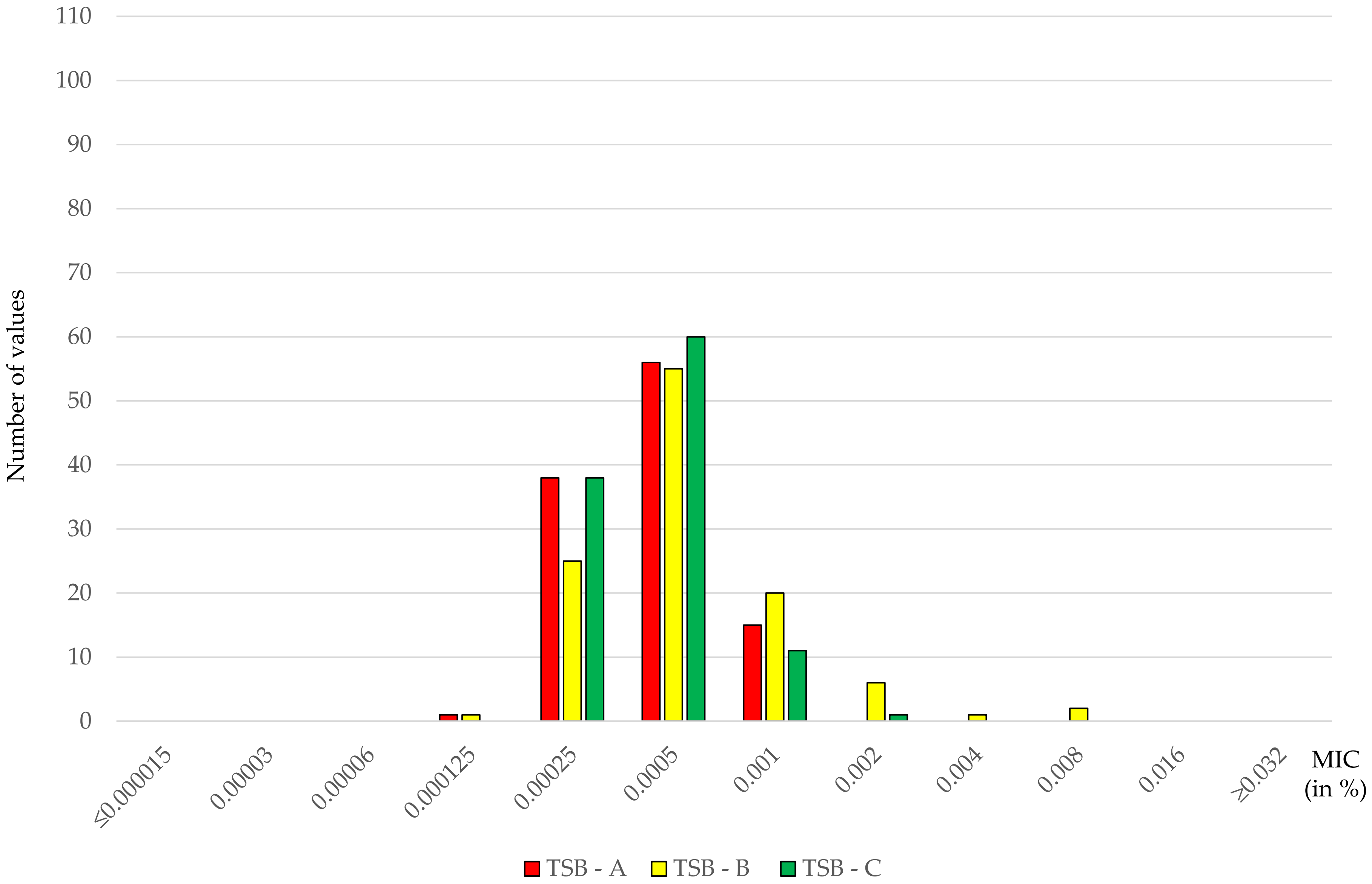

Figure S4d: Differences of the media lots for *P. aeruginosa* ATCC® 15442 and octenidine
