# Supplementary material for: Development of Quality Control Ranges for Biocide Susceptibility Testing
Source: Pathogens. 2022 Feb 8;11(2):223. doi: 10.3390/pathogens11020223 (PMC8878709; doi:10.3390/pathogens11020223)
Supplement: Supplementary file 1 [file pathogens-11-00223-s001.zip › pathogens-1547182-supplementary/Table S1.pdf]

Table S1. Summary of the QC ranges of *S. aureus* ATCC® 6538

MIC (in %)	Benzalkonium chloride	Chlorhexidine	Polyhexanide	Octenidine
0.064				
0.032				
0.016				
0.008				
0.004				
0.002	3		5	
0.001			18	
0.0005			38	1
0.00025	4	3	90	86
0.000125	127	126	167	194
0.00006	195	196	12	48
0.00003	1	5		1
0.000015				
0.000008				

Biocide concentrations not tested are displayed in gray. The respective QC ranges are displayed as boxes
