# Supplementary material for: Development of Quality Control Ranges for Biocide Susceptibility Testing
Source: Pathogens. 2022 Feb 8;11(2):223. doi: 10.3390/pathogens11020223 (PMC8878709; doi:10.3390/pathogens11020223)
Supplement: Supplementary file 1 [file pathogens-11-00223-s001.zip › pathogens-1547182-supplementary/Table S2.pdf]

Table S2. *E. hirae* ATCC® 10541

a) Benzalkonium chloride

MIC (in %)	Lab 1	Lab 2	Lab 3	Lab 4	Lab 5	Lab 6	Lab 7	Lab 8	Lab 9	Lab 10	Lab 11
≥0.032											
0.016											
0.008											
0.004											
0.002											
0.001											
0.0005						1				1	
0.00025	28	17	23	27	7	20		28	30	17	27
0.000125	2	13	7	3	23	9	30	2		12	3
0.00006											
0.00003											
0.000015											
≤0.000008											

The white area represents the proposed QC range. The gray areas represent the parts of the test range outside the QC range.

b) Chlorhexidine

MIC (in %)	Lab 1	Lab 2	Lab 3	Lab 4	Lab 5	Lab 6	Lab 7	Lab 8	Lab 9	Lab 10	Lab 11
≥0.016											
0.008											
0.004											
0.002											
0.001											
0.0005											
0.00025	5			5		1		2	1		5
0.000125	9	9	21	14	8	7	1	4	12	17	19
0.00006	16	21	9	11	22	22	29	23	17	13	6
0.00003								1			
0.000015											
≤0.000008											

The white area represents the proposed QC range. The gray areas represent the parts of the test range outside the QC range.

c) Polyhexanide

MIC (in %)	Lab 1	Lab 2	Lab 3	Lab 4	Lab 5	Lab 6	Lab 7	Lab 8	Lab 9	Lab 10	Lab 11
≥0.064											
0.032											
0.016											
0.008							*1*				
0.004							*1*				*1*
0.002				7		1	7	3			8
0.001	10	1	8	8	2	3	11	2	1	10	11
0.0005	13	8	11	8	9	10	6	2	18	15	10
0.00025	7	19	10	7	11	16	4	21	10	5	
0.000125		2	1		8			2	1		
0.00006											
0.00003											
≤0.000015											

The white area represents the proposed QC range. The gray areas represent the parts of the test range outside the QC range. Values outside this QC range are displayed in red and italics.

d) Octenidine

MIC (in %)	Lab 1	Lab 2	Lab 3	Lab 4	Lab 5	Lab 6	Lab 7	Lab 8	Lab 9	Lab 10	Lab 11
≥0.032											
0.016											
0.008											
0.004											
0.002											
0.001											*2*
0.0005			2	1						1	9
0.00025	15	6	12	11	5	8		6	5	13	14
0.000125	15	24	16	18	24	22	21	24	25	16	5
0.00006					1		9				
0.00003											
≤0.000015											

The white area represents the proposed QC range. The gray areas represent the parts of the test range outside the QC range. Values outside this QC range are displayed in red and italics.
