# Supplementary material for: Development of Quality Control Ranges for Biocide Susceptibility Testing
Source: Pathogens. 2022 Feb 8;11(2):223. doi: 10.3390/pathogens11020223 (PMC8878709; doi:10.3390/pathogens11020223)
Supplement: Supplementary file 1 [file pathogens-11-00223-s001.zip › pathogens-1547182-supplementary/Table S3.pdf]

Table S3. Summary of the QC ranges of *E. hirae* ATCC® 10541

MIC (in %)	Benzalkonium chloride	Chlorhexidine	Polyhexanide	Octenidine
0.064				
0.032				
0.016				
0.008			1	
0.004			2	
0.002			26	
0.001			67	2
0.0005	2		110	13
0.00025	224	19	110	95
0.000125	104	121	14	210
0.00006		189		10
0.00003				
0.000015				
0.000008				

Biocide concentrationsnot tested are displayed in gray. The respective QC ranges are displayed as boxes
