# Supplementary material for: Development of Quality Control Ranges for Biocide Susceptibility Testing
Source: Pathogens. 2022 Feb 8;11(2):223. doi: 10.3390/pathogens11020223 (PMC8878709; doi:10.3390/pathogens11020223)
Supplement: Supplementary file 1 [file pathogens-11-00223-s001.zip › pathogens-1547182-supplementary/Table S4.pdf]

Table S4. Summary of the QC ranges of *E. coli* ATCC® 10536

MIC (in %)	Benzalkonium chloride	Chlorhexidine	Polyhexanide	Octenidine
0.064			1	
0.032				
0.016				
0.008				
0.004				
0.002	25		4	1
0.001	217	1	7	6
0.0005	87	5	41	35
0.00025	1	47	113	118
0.000125		52	157	141
0.00006		141	7	29
0.00003		84		
0.000015				
0.000008				

Biocide concentrationsnot tested are displayed in gray. The respective QC ranges are displayed as boxes
