# Supplementary material for: Development of Quality Control Ranges for Biocide Susceptibility Testing
Source: Pathogens. 2022 Feb 8;11(2):223. doi: 10.3390/pathogens11020223 (PMC8878709; doi:10.3390/pathogens11020223)
Supplement: Supplementary file 1 [file pathogens-11-00223-s001.zip › pathogens-1547182-supplementary/Table S5.pdf]

Table S5. *P. aeruginosa* ATCC® 15442

a) Benzalkonium chloride

MIC (in %)	Lab 1	Lab 2	Lab 3	Lab 4	Lab 5	Lab 6	Lab 7	Lab 8	Lab 9	Lab 10	Lab 11
≥0.032				*1*							
0.016											
0.008	10	7	10	8	8	8	8	12	10	10	10
0.004	20	23	20	21	22	22	21	18	20	20	20
0.002							1				
0.001											
0.0005											
0.00025											
0.000125											
0.00006											
0.00003											
0.000015											
≤0.000008											

The white area represents the proposed QC range. The gray areas represent the parts of the test range outside the QC range. Values outside this QC range are displayed in red and italics.

b) Chlorhexidine

MIC (in %)	Lab 1	Lab 2	Lab 3	Lab 4	Lab 5	Lab 6	Lab 7	Lab 8	Lab 9	Lab 10	Lab 11
≥0.016				2	4	3					
0.008	5	2	6		9	14			2	6	5
0.004	19	16	16	9	10	10	8	28	26	20	16
0.002	6	10	8	14	5	3	15	2	2	4	7
0.001		2		4	2		5				2
0.0005				1			2				
0.00025											
0.000125											
0.00006											
0.00003											
0.000015											
≤0.000008											

The white area represents the range calculated by the RangeFinder software. The gray areas represent the parts of the test range outside the calculated QC range. Due to the lack of an exact upper limit no QC range was set.

c) Polyhexanide

MIC (in %)	Lab 1	Lab 2	Lab 3	Lab 4	Lab 5	Lab 6	Lab 7	Lab 8	Lab 9	Lab 10	Lab 11
≥0.064	10			4	4	11	5		20		5
0.032	2	5	4		8	2	2			8	13
0.016	8	15	13	13	8	4	13	10		11	2
0.008	7	1	3	8	4	6		4	8	3	9
0.004	3	8	9	4	6	6	8	13	2	8	1
0.002		1	1	1		1	2	3			
0.001											
0.0005											
0.00025											
0.000125											
0.00006											
0.00003											
≤0.000015											

The white area represents the range calculated by the RangeFinder software. The gray areas represent the parts of the test range outside the calculated QC range. Due to the lack of an exact upper limit no QC range was set.

d) Octenidine

MIC (in %)	Lab 1	Lab 2	Lab 3	Lab 4	Lab 5	Lab 6	Lab 7	Lab 8	Lab 9	Lab 10	Lab 11
≥0.032											
0.016											
0.008		*1*				*1*					
0.004		*1*									
0.002				3	1						3
0.001	3	2	10	6	5	2		2	4	2	10
0.0005	20	16	13	16	12	16	3	23	22	20	10
0.00025	7	10	7	5	12	10	26	5	4	8	7
0.000125						1	1				
0.00006											
0.00003											
≤0.000015											

The white area represents the proposed QC range. The gray areas represent the parts of the test range outside the QC range. Values outside this QC range are displayed in red and italics.
