# Supplementary material for: Development of Quality Control Ranges for Biocide Susceptibility Testing
Source: Pathogens. 2022 Feb 8;11(2):223. doi: 10.3390/pathogens11020223 (PMC8878709; doi:10.3390/pathogens11020223)
Supplement: Supplementary file 1 [file pathogens-11-00223-s001.zip › pathogens-1547182-supplementary/Table S6.pdf]

Table S6. Summary of the QC ranges of *P. aeruginosa* ATCC® 15442

MIC (in %)	Benzalkonium chloride	Chlorhexidine	Polyhexanide	Octenidine
0.064			59	
0.032	1		44	
0.016		9	97	
0.008	101	49	53	2
0.004	227	178	68	1
0.002	1	76	9	7
0.001		15		46
0.0005		3		171
0.00025				101
0.000125				2
0.00006				
0.00003				
0.000015				
0.000008				

Biocide concentrationsnot tested are displayed in gray. The respective QC ranges are displayed as boxes
